# DNA methylation analysis of paediatric low-grade astrocytomas identifies a tumour-specific hypomethylation signature in pilocytic astrocytomas

**DOI:** 10.1186/s40478-016-0323-6

**Published:** 2016-05-27

**Authors:** Jennie N. Jeyapalan, Gabriel T. Doctor, Tania A. Jones, Samuel N. Alberman, Alexander Tep, Chirag M. Haria, Edward C. Schwalbe, Isabel C. F. Morley, Alfred A. Hill, Magdalena LeCain, Diego Ottaviani, Steven C. Clifford, Ibrahim Qaddoumi, Ruth G. Tatevossian, David W. Ellison, Denise Sheer

**Affiliations:** Blizard Institute, Barts and The London School of Medicine and Dentistry, Queen Mary University of London, 4 Newark Street, London, E1 2AT UK; Northern Institute for Cancer Research, Newcastle University, Newcastle upon Tyne, UK; Department of Applied Sciences, Northumbria University, Newcastle upon Tyne, UK; Department of Oncology, St Jude Children’s Research Hospital, Memphis, TN USA; Department of Pathology, St Jude Children’s Research Hospital, Memphis, TN 38105-3678 USA

**Keywords:** Diffuse astrocytomas, MAPK pathway, AP-1 targets, FOS, Cyclin D1, Enhancers

## Abstract

Low-grade gliomas (LGGs) account for about a third of all brain tumours in children. We conducted a detailed study of DNA methylation and gene expression to improve our understanding of the biology of pilocytic and diffuse astrocytomas. Pilocytic astrocytomas were found to have a distinctive signature at 315 CpG sites, of which 312 were hypomethylated and 3 were hypermethylated. Genomic analysis revealed that 182 of these sites are within annotated enhancers. The signature was not present in diffuse astrocytomas, or in published profiles of other brain tumours and normal brain tissue. The AP-1 transcription factor was predicted to bind within 200 bp of a subset of the 315 differentially methylated CpG sites; the AP-1 factors, FOS and FOSL1 were found to be up-regulated in pilocytic astrocytomas. We also analysed splice variants of the AP-1 target gene, *CCND1*, which encodes cell cycle regulator cyclin D1. *CCND1a* was found to be highly expressed in both pilocytic and diffuse astrocytomas, but diffuse astrocytomas have far higher expression of the oncogenic variant, *CCND1b*. These findings highlight novel genetic and epigenetic differences between pilocytic and diffuse astrocytoma, in addition to well-described alterations involving *BRAF, MYB* and *FGFR1*.

**Electronic supplementary material:**

The online version of this article (doi:10.1186/s40478-016-0323-6) contains supplementary material, which is available to authorized users.

## Introduction

Pilocytic astrocytomas (WHO grade I) constitute the majority of paediatric low-grade gliomas (LGGs). They usually arise in the cerebellum but are also found in other sites such as the optic pathways and cerebral hemispheres. Pilocytic astrocytomas typically contain a *BRAF* fusion but occasionally a *BRAF*^*V600E*^ mutation, *RAF1* fusion, intragenic duplication of *FGFR1*, or other rarer alterations are present [21, 65]. Diffuse astrocytomas (WHO grade II) usually occur in the cerebral hemispheres, but are also found in the brainstem and spinal cord. Various genetic alterations have been identified in diffuse astrocytomas, including *BRAF*^*V600E*^ mutations, intragenic duplication of *FGFR1*, structural alterations of the *MYB* oncogene and gene fusions involving *FGFR1, FGFR3*, *MYB* and *MYBL1* [65]. Virtually all the key genetic alterations in pilocytic and diffuse astrocytomas give rise to constitutive activation of the ERK/MAPK pathway [10, 20, 43, 65], but these tumour types exhibit significant biological and clinical heterogeneity. While pilocytic astrocytomas are well-circumscribed, non-invasive tumours, diffuse astrocytomas invade surrounding tissue and thus have a worse outcome. It is therefore likely that other factors, such as epigenetics and regulating RNAs, as well as the cell of origin and the developing brain environment, influence the divergent phenotypic behaviour [1, 22]. DNA methylation is altered in both cancer [14] and during brain development [30]. However, its contribution to paediatric low-grade glioma tumorigenesis has not been extensively studied.

We have conducted a comprehensive analysis of DNA methylation together with gene expression in pilocytic and diffuse astrocytomas from two independent tumour sets (test set *n* = 27 and validation set *n* = 59) using the Illumina HumanMethylation450 BeadChip (450K). Comparison between the tumour types and normal brain controls identified tumour-specific differences in the DNA methylation patterns. We identified a hypomethylation signature specific to pilocytic astrocytomas, characterised by differentially methylated CpG sites predominantly in annotated enhancers. Additionally, we were able to show that the AP-1 transcription factor complex is predicted to bind at a number of the differentially methylated CpG sites. The AP-1 complex is formed by dimerisation of the FOS and JUN family of transcription factors and is activated by the MAPK pathway [9, 40]. In this study, the FOS family of transcription factors was found to be up-regulated in pilocytic astrocytomas. The AP-1 gene target *CCND1a* was also up-regulated in both pilocytic and diffuse astrocytomas, with higher levels of the oncogenic *CCND1b* transcript expressed in the diffuse astrocytomas.

## Materials and methods

### Low-grade astrocytoma cohort

The test tumour set consisted of 17 pilocytic astrocytomas and 10 diffuse astrocytomas (Additional file 1: Table S1). Validation tumour set 1 consisted of 23 pilocytic astrocytomas and 8 diffuse astrocytomas and validation tumour set 2 consisted of 45 pilocytic astrocytomas; 6 diffuse astrocytomas and 8 oligoastrocytomas (Additional file 1: Table S1). All tumours were obtained as surgical specimens. Ages of the patients at diagnosis ranged from 3 to 20 years. Access to tumours and linked clinical data was given in accordance with Institutional Review Board and MREC regulations: St Jude Children’s Research Hospital (USA) XPD07-107/IRB; Newcastle (UK) REC ref No 2002/112; Blizard Institute (UK) ICMS/PR/09/77. The controls were human neural progenitor cells (ReN VM cell-line), adult brain, foetal cerebellum, foetal frontal lobe and foetal brain (normal brain, BioChain).

### Infinium HumanMethylation450 BeadChip processing

Sample DNA (1 μg) was bisulphite-converted using the EZ DNA methylation kit (Zymo Research) and analysed using the Infinium HumanMethylation450 BeadChip (Illumina Inc.). The samples and 450K BeadChips were processed according to the manufacturer’s protocol at Barts and The London Genome Centre, UK. Pre-processing of the 450K dataset was performed using Genome studio software v.2011.1 (Illumina Inc.). Quality control of bisulphite conversion was performed by calculating the ratio of unmethylated probe to methylated probe. Samples that had incomplete conversion (a ratio >0.2) were removed. Methylation status for each probe is given as a Beta value (β-value). The β-value is the ratio of the methylated probe intensity and the overall intensity (sum of the methylated and unmethylated probe intensities). Pre-processing of the data was then performed using R (version 2.15.0). Peak correction was performed [39] and probes that contained a minor allele frequency of >5 % within 50 bp of the target site were removed [59]. The Illumina annotation [3] and an enhanced annotation ([42] were added to the peak-corrected datasets (Additional file 2: Table S2). We excluded probes located on the X- and Y- chromosomes from further analysis. The dataset generated in this study has been deposited in the Gene Expression Omnibus (GEO) under accession GSE77241.

### Differential methylation analysis

Differential methylation analysis was performed using the MethLAB R-based programme [23] (R version 2.15.0). The programme enables us to identify significantly differentially methylated CpGs from the corrected β-values. The linear model with the factor of interest (tumour type – pilocytic, diffuse, control) was computed, with other varying factors: bead chip number (1–5), *BRAF* status (fusion, V600E mutation, WT), sample location (infratentorial/supratentorial), age group (foetal/HNSC, <3 years, > = 3 years, >16 years) and gender included. A class covariance and FDR correction (Benjamini-Hochberg) were performed. In this analysis the dependent variable is the β-value for each probe, and the independent variables are phenotypic factors such as tumour type. Further details for each analysis are shown in Additional file 3: Supplementary Methods. The differentially methylated CpG sites of interest had a differential change (delta Beta value) of ≥0.3 with FDR-corrected *p*-value <0.05. A list of the comparisons performed is shown in Additional file 4: Table S3.

### Expression analysis

Expression analysis was performed on 8 pilocytic astrocytomas and 10 diffuse astrocytomas from the test tumour set, using Affymetrix Human U133_plus2 arrays [65]. The data were analysed using GeneSpring software (Agilent). Analysis was performed using the RMM model, with median baseline correction and data log2 transformation (Additional file 5: Table S4). For differential expression analysis, the tumour groups were averaged and the difference between the two groups was taken. Differentially expressed genes had a fold change of >2(differential log2 transformed values >1).

### Pathway analysis and transcription factor binding motif analysis

Ingenuity pathway analysis (Ingenuity Systems Inc.) was used to identify key signalling and biological pathways from the genes identified as differentially methylated. For the top biological functions and canonical pathways, the log *p*-value was calculated by Fisher exact Test, with a threshold set at a *p*-value <0.05. The TFsearch software which utilises the TRANSFAC database [15] was used to predict transcription factor binding sites 100 bp either side of the differentially methylated CpG sites.

### Marmal-aid database analysis

To analyse the differentially methylated CpG sites in published profiles for other brain tumours, we used the Marmal-aid database [33]. Hierarchical clustering and heatmaps were produced using the R-programme fastcluster on R version 2.15.0. The database was used to assess 450K methylation datasets for adult low-grade gliomas (TCGA), adult and paediatric glioblastomas (GSE36278 [51]) and pilocytic astrocytomas (GSE41826, [27]). Heatmaps were also produced for validation tumour set 2 and for medulloblastomas [45].

### Bisulphite converted DNA and PCR for pyrosequencing

Quantitative DNA methylation analysis was performed at 7 selected genes using pyrosequencing in the test tumour set and in validation set 2 (Additional file 6: Table S5). Amplification of 50 ng of bisulphite-converted DNA was performed using 0.03 U/μl Platinum Taq polymerase (Life Technologies), 1 x PCR buffer, 3 mM MgCl_2_, 0.8 mM dNTP, 200 nM forward primer, 200 nM reverse primer, 0.5 M Betaine (Sigma Aldrich) and nuclease-free water. PCR cycling at 92 °C for 10 mins, then 44 cycles at 92 °C for 30 s, 50 °C for 1 min and 72 °C for 40 s, with a final extension step of 72 °C for 5 mins. Strand separation and pyrosequencing were performed at Barts and The London Genome Centre, UK.

Methylation analysis of 11 CpG sites in the *CCND1* gene and analysis of the G870A SNP were performed by amplification of 50 ng of bisulphite converted DNA as stated above. The primers amplified a region of 362 bp encompassing exon 4 and the intron boundary (Primers, forward 5′-GTTTTAGATGTGAAGTTTATTTTTAA-3′ and reverse 5′-TATAAAAACCTCCCAACCAATC-3′). The amplicons were Sanger sequenced in both directions to obtain CpG and SNP status.

### CCND1 qPCR assay

Conversion of 500 ng of RNA into cDNA was performed using the SuperScript II reverse transcriptase (Life Technologies) following the manufacturer’s protocol. Amplification was performed in duplicate with a technical replicate (*n* = 2 for cDNA conversion). PCR reactions consisted of SYBR® Green Jumpstart Taq ReadyMix ^TM^ (1x; Sigma‐Aldrich), forward and reverse primers (10 μM each), made up to 20 μl with nuclease-free water. Primer sequences for *CCND1a* (forward – 5′-CTCTCCAGAGTGATCAAGTGTGACCC-3′, reverse – 5′-TGTGCAAGCCAGGTCCACC-3′;[7]; *CCND1b* (forward- 5′-AACAGATCATCCGCAAACACGC-3′, reverse – 5′-CATGAGTCCTTCCACGATACC-3′; [5]; GAPDH (forward- 5′‐GTGAACCATGAGAAGTATGACAAC‐3′, reverse- 5′‐ CATGAGTCCTTCCACGATACC‐3′) and TBP (forward - 5′‐CACGAACCACGGCACTGATT‐3′, reverse 5′‐TTTTCTTGCTGCCAGTCTGGAC‐3′). The cycling conditions were 50 °C for 2 min, 95 °C for 10 min, followed by cycles of 95 °C for 15 s, 60 °C for 40 s and 72 °C for 40 s, for 40 cycles. A dissociation stage of 95 °C for 15 s, 60 °C for 1 min, 95 °C for 15 s and 60 °C for 15 s, was included. PCRs were performed on the 7500 Real-Time PCR system (Applied Biosystems®). The fold change was calculated using the ΔΔC_T_ relative quantification method [32]**.**

### MicroRNA Taqman assays

Conversion of 10 ng of RNA into cDNA was performed using the Taqman^®^ MicroRNA Reverse Transcription (RT) kit (Life Technologies) following the manufacturer’s protocol. RT-reaction conditions were 1 x Reverse Transcription buffer, 1 mM dNTP, 3.3 U/μl MultiScribe^™^ Reverse Transcriptase, 0.25 U/μl RNase Inhibitor, 10 ng RNA sample, 1 x RT microRNA-specific primer and nuclease-free water. RT-reaction was incubated at 16 °C for 30 mins, 42 °C for 30 mins and 85 °C for 5 mins. TaqMan^®^ miRNA assays (Life Technologies) were used to analyse expression levels of microRNAs miR-21* and miR-155, with all samples normalised to control microRNA, miR-423-3p. PCR reaction conditions were 1 ng/μl RT-product, 1x Taqman^®^ Universal PCR master mix II (no UNG), 1 x Taqman^®^ probe and nuclease-free water. PCR cycling conditions were 95 °C for 10 mins, followed by 40 cycles of 95 °C for 15 s and 60 °C for 1 min. Fold changes were calculated relative to the average expression in adult and foetal cerebellum and frontal lobe (*n* = 7).

## Results

### Identification of a hypomethylation signature specific to pilocytic astrocytomas

DNA methylation profiles were identified for the test tumour set (11 infratentorial pilocytic astrocytomas; 6 supratentorial pilocytic astrocytomas and 10 diffuse astrocytomas), 4 normal brain control samples and the ReN VM neural stem cell-line using the Illumina Infinium HumanMethylation450 BeadChip [3, 8]. Profiles were then subjected to extensive comparisons, as summarised in Additional file 4: Table S3.

**Fig. 1:**
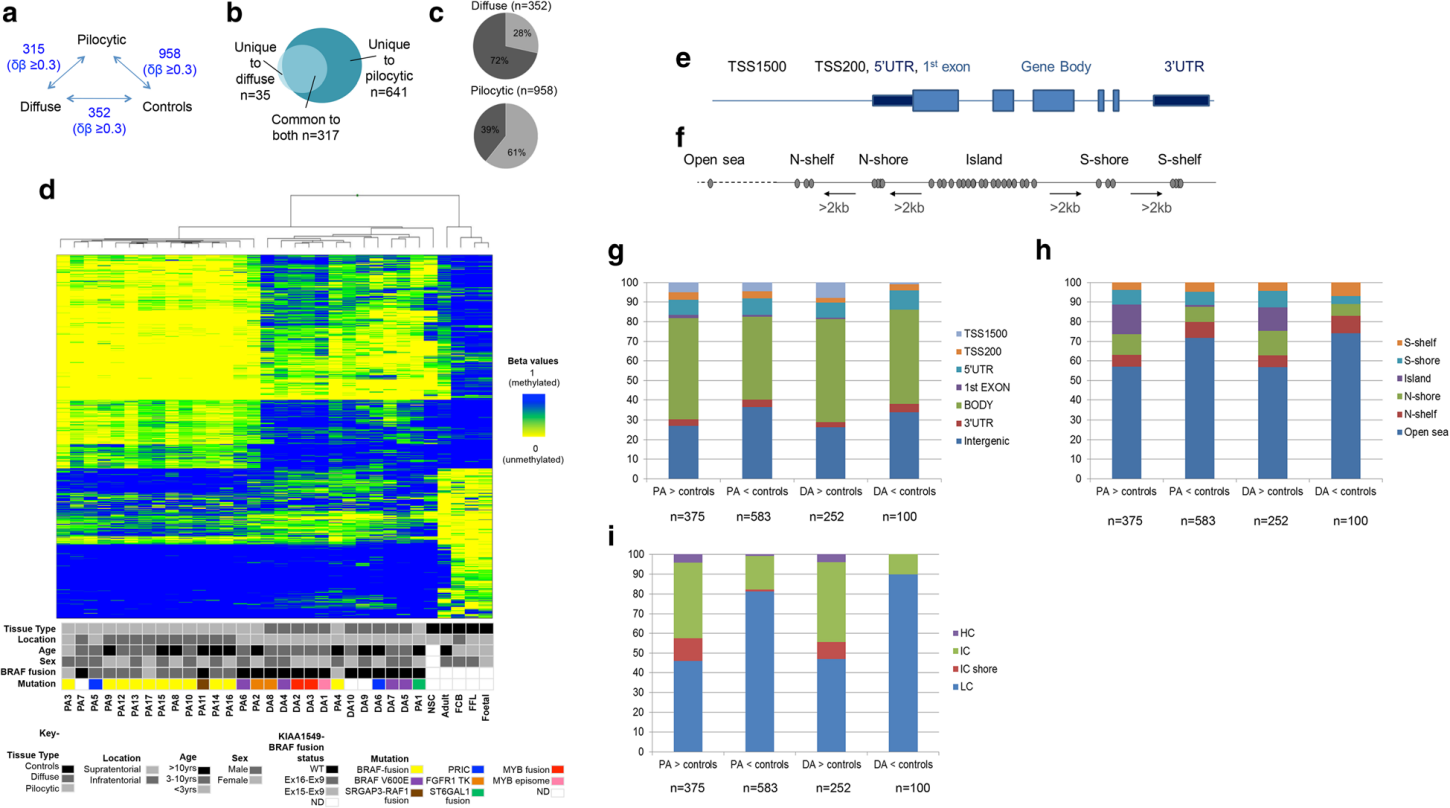
Features of 993 differentially methylated CpG sites in pilocytic and diffuse astrocytomas compared to controls. **a** Three-way comparison of differentially methylated sites in pilocytic astrocytomas, diffuse astrocytomas and control brain tissue. **b** Numbers of common and unique differentially methylated sites in pilocytic and diffuse astrocytomas. **c** Percentages of hypermethylated (dark grey) and hypomethylated (light grey) differentially methylated sites in pilocytic and diffuse astrocytomas compared to normal controls. **d** Hierarchical clustered heatmap showing 993 differentially methylated sites. Methylation beta values are represented in shades of blue = 0 (methylated) to yellow = 1 (unmethylated). Tumour type, tumour location, age, sex, *KIAA1549-BRAF* fusion status, and genomic mutations are shown [65]. FFL, Foetal frontal lobe; Foetal, foetal brain; FCB, foetal cerebellum; Adult, adult brain, NSC, neural stem cells; PA, pilocytic astrocytomas; DA, diffuse astrocytomas; ND, not determined. **e** Diagrammatic representation of the Illumina 450K annotated CpG features in relation to intragenic locations, and **f** in relation to UCSC Genome Browser-annotated CpG islands. **g** Distribution of the differentially methylated CpG sites across the annotated gene regions. TSS1500, 201-1500 bp upstream of transcription start (TSS); TSS200, up to 200 bp upstream of the TSS; UTR, untranslated region. **h** Distribution of the differentially methylated CpG sites in relation to CpG islands. **i** Distribution of the differentially methylated CpG sites in relation to CpG density [42]. HC, high CpG density; IC, intermediate CpG density; IC shore, intermediate CpG density shore; LC, low CpG density

We first conducted a three-way comparison of pilocytic astrocytomas, diffuse astrocytomas and normal brain and ReN VM neural stem cell-line controls (Fig. 1a; Additional file 4: Table S3, Panel A and Additional file 7: Table S6). Differential methylation was identified at 958 CpG sites in pilocytic astrocytomas vs. controls and 352 sites in diffuse astrocytomas vs. controls (FDR-corrected *p*-value <0.05 and Δβ >0.3). Of the 993 differentially methylated CpG sites found in either of these comparisons, 317 sites were common to both comparisons (Fig. 1b, c). Ingenuity Pathway Analysis of genes associated with the 993 sites is shown in Additional file 8: Table S7. We also performed differential methylation analysis comparing the genetic alteration status, specifically looking at *BRAF* fusion status (*n* = 12) and then grouped *BRAF* fusion and *BRAF*^*V600E*^ status (*n* = 17) of the tumours (Additional file 3: Supplementary Methods). No significant CpG sites were identified.

**Fig. 2:**
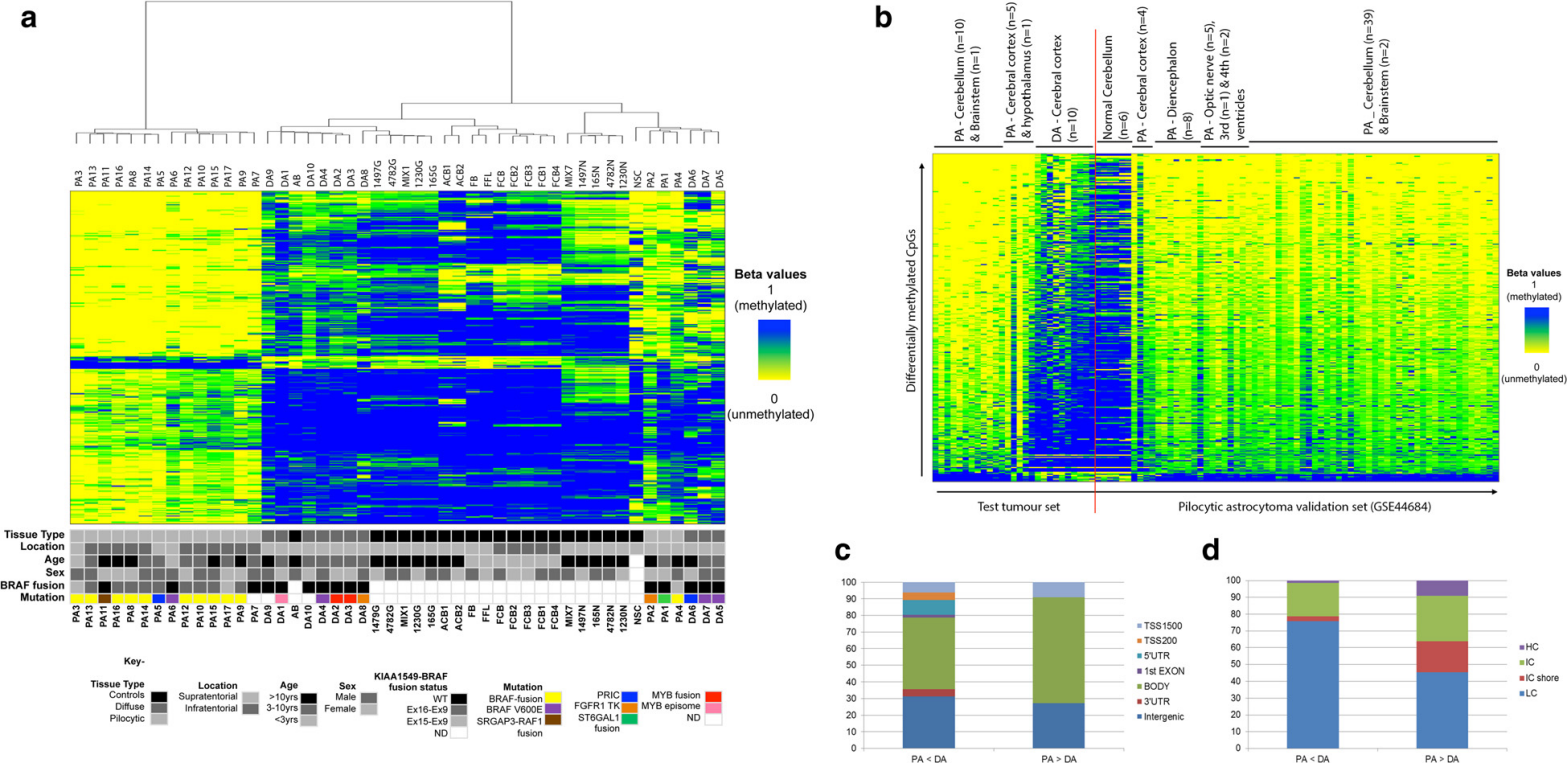
Differentially methylated CpG sites in pilocytic and diffuse astrocytomas: comparison with normal brain tissue, and characteristics. **a** Hierarchical clustering of pilocytic and diffuse astrocytomas, normal controls, and additional brain samples. Test tumours: PA, pilocytic astrocytomas; DA, diffuse astrocytomas. Controls: FFL, Foetal frontal lobe; FB, foetal brain; FCB, foetal cerebellum; AB, adult brain, ACB, adult cerebellum; NSC, neural stem cells. Additional datasets from normal brain tissue are derived from GEO Accession GSE41826: 4782 N/4782G, 165 N/165G, 1230 N/1230G, 1497 N/1497G, neuronal or glial component of cerebral cortex; MIX1 and MIX7, mix of glial and neuronal components of cerebral cortex [12]. The overwhelming majority of hypomethylated sites are specific to pilocytic astrocytomas. Two main branches emerge, one of which contains most of the pilocytic astrocytomas and the other contains the normal brain controls and diffuse astrocytomas, which grouped with glial component of the cerebral cortex. Foetal brain regions and adult cerebellum clustered together. A subset of astrocytomas grouped with the neural stem cells. Columns represent individual samples; rows represent 450K CpG probes. As four values were missing in the normal brain dataset only 311/315 sites are shown. Methylation beta values are represented in shades of yellow = 0 (unmethylated) to blue = 1 (methylated). **b** Validation of the hypomethylated signature in a larger published set of pilocytic astrocytomas (PA validation set 2; *n* = 61; GSE44684 [27]). The hypomethylation signature was identified in both infratentorial and supratentorial pilocytic astrocytomas. BRAF-fusions were present in 51/62 samples in this tumour set. Only 287/315 sites were present in the published data set. Tumour samples on the left of the red line are the test tumours in the current study, samples on the right of the line are validation set. Columns represent individual samples; rows 450K CpG probes. **c** Distribution of the 315 differentially methylated CpG sites across the Illumina 450K-annotated location CpG features. TSS1500, 201-1500 bp upstream of transcription start (TSS); TSS200, up to 200 bp upstream of the TSS; UTR, untranslated region **d** Distribution of the 315 differentially methylated sites in regions of high, intermediate and low CpG density [42]. HC, high CpG density; IC, intermediate CpG density; IC shore, intermediate CpG density shore; LC, low CpG density

Hierarchical cluster analysis of the samples at the 993 CpG sites showed clustering of all the pilocytic astrocytomas except two supratentorial tumours, PA1 and PA4, which clustered with the diffuse astrocytomas and neural stem cell sample (Fig. 1d). Whilst PA4 contained the *BRAF* fusion, PA1 contained the STGAL1-WHSC1 fusion together with further complex rearrangements as shown by whole genome sequencing [65], which may account for this tumour clustering with the diffuse astrocytomas. We then examined the locations of the 993 sites relative to CpG islands and to different regions of the 536 genes associated with 671 of these sites (Fig. 1e,f,g,h,i). Unlike other cancers where changes are mainly identified in the shores [19], the majority of the differentially methylated CpGs were found to be located in open sea regions. The sites associated with genes were mostly located within the gene body and regions up- or down-stream of the gene, rather than at transcription start sites. As the unsupervised clustering of the total 450K dataset could not distinguish between the two tumour groups we compared pilocytic astrocytomas vs. diffuse astrocytomas. Only 315 differentially methylated sites were identified after exclusion of differences between the matched brain locations of the tumours. The majority of these sites are hypomethylated in pilocytic astrocytomas and the distinctive signature was not present in normal brain controls (Fig. 2). The lack of CpG sites which are differentially methylated in diffuse astrocytomas could be due to the genetic heterogeneity within the diffuse astrocytromas but may also be due to normal brain admixture in some of the tumour samples, which can be more of a feature of diffuse astrocytomas than the well-circumscribed pilocytic astrocytomas.

We confirmed our findings by showing good correlation between the 450K BeadChip values with pyrosequencing methylation values for 5 CpG sites (Additional file 3: Figure S1). We also validated the methylation status at selected genes using pyrosequencing on the test tumour set and validation tumour set 1. The genes chosen for validation showed differential methylation between pilocytic astrocytomas and normal brain controls or between pilocytic astrocytomas and diffuse astrocytomas. The genes of interest were the RAS-RAF inhibitor, *SPRED2* (Additional file 3: Figure S2; [60, 61]); *miR-21,* an AP-1 target which is involved in gliomagenesis (Additional file 3: Figures S3 and S5; [11, 55, 56]); *miR-155*, which is involved in inflammation and the mTOR pathway (Additional file 3: Figures S4 and S5 [37, 62]), and two genes involved in drug resistance, *ABCC3* (Additional file 3: Figure S6 [4, 67]) and *SELENBP1* (Additional file 3: Figure S7; [18, 47, 66]).

The 315 CpG sites which were predominately hypomethylated in pilocytic astrocytomas were validated in a second set of paediatric low-grade astrocytomas, which were analysed independently with the Illumina 450K system (validation set 2, *n* = 59; Additional file 3: Figure S8). Additionally, the differences were confirmed to be irrespective of tumour location, as the hypomethylation signature was also present in pilocytic astrocytomas located in the brain stem, diencephalon and optic nerve (Fig. 2b; published pilocytic astrocytoma validation set 3, *n* = 61; GSE44684 [27]). To confirm that the distinctive signature was specific for pilocytic astrocytomas, the 315 sites were then examined in published findings of DNA methylation in other brain tumours, using the Marmal-aid database [33]. The hypomethylation signature was not present in other low-grade astrocytomas (adult gliomas –TCGA**)**, paediatric and adult glioblastomas (GSE36278 [51]), medulloblastomas [45] or ependymomas (GSE45353 [35]; Additional file 3: Figures S9 and S10; Additional file 9: Table S8). The signature was also absent in tissue from foetal brain tissue (GSE58885 [49]**;** Additional file 3: Figure S11).

We then examined the genomic features of the 315 differentially methylated sites and found that 235 sites (75 %) were in regions of low CpG density, and 182 sites (58 %) were in enhancer regions (Illumina annotated, statistically significant - Chi-square *p*-value <0.01; 149/182 sites were in regions of low CpG density; (Additional file 10: Table S9). Ingenuity Pathway Analysis of the 185 genes that were directly linked to 217/315 sites showed significant association with *Cancer* (Additional file 11: Table S10). The remaining 98 CpG sites were located in intergenic regions. We assigned 82 genes to these intergenic sites by finding the closest transcription start sites (TSS) (Additional file 10: Table S9). Differential gene expression of ≥ 2-fold between pilocytic and diffuse astrocytomas was found for 56/267 genes (185 annotated genes and 82 closest TSS-assigned genes; Additional file 12: Table S11).

**Fig. 3:**
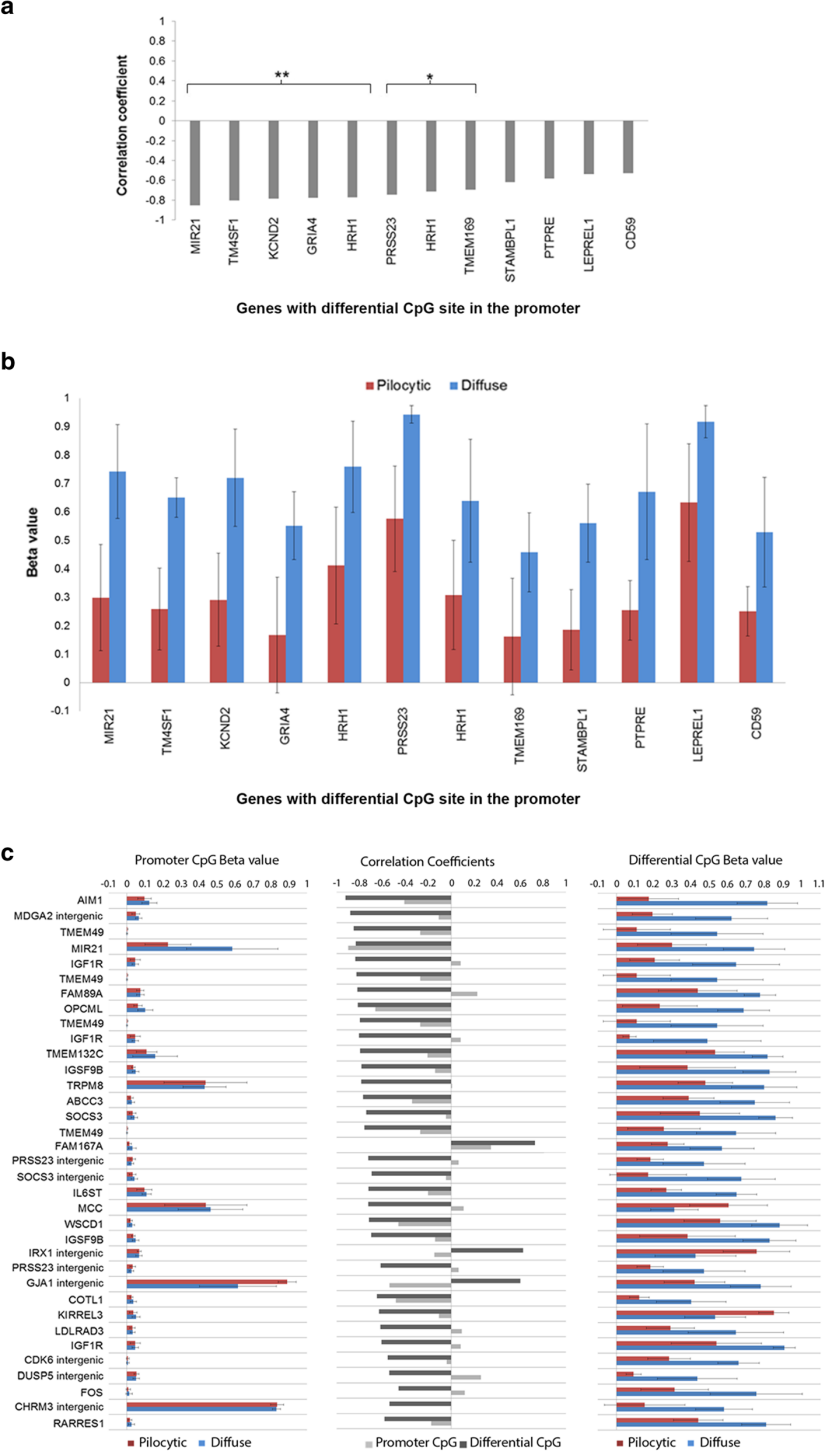
Correlation between gene expression and the differentially methylated CpG sites for genes showing ≥2 fold difference in expression between pilocytic and diffuse astrocytomas. **a** Correlation coefficients for differential CpG sites located within the promoter region. CpG sites that showed significant correlation with gene expression are highlighted (** *p*-value <0.01, * *p*-value <0.05). **b** Methylation status (beta values) for the pilocytic and diffuse astrocytomas for the promoter differential CpG site. **c** Correlation coefficients for differential CpG sites located within the gene body and intergenic regions that showed significant correlation with gene expression (*p*-value <0.05). Genes showing the greatest statistical significance are placed at the top. Correlation coefficients for corresponding promoter CpG sites, and the methylation status (Beta values) for the promoter CpG site and the differentially methylated CpG site are also shown. Pilocytic astrocytomas (*n* = 8, 4 supratentorial and 4 infratentorial tumours), Diffuse astrocytomas (*n* = 10). Error bars show the standard deviation for the average beta value for each tumour group

**Fig. 4:**
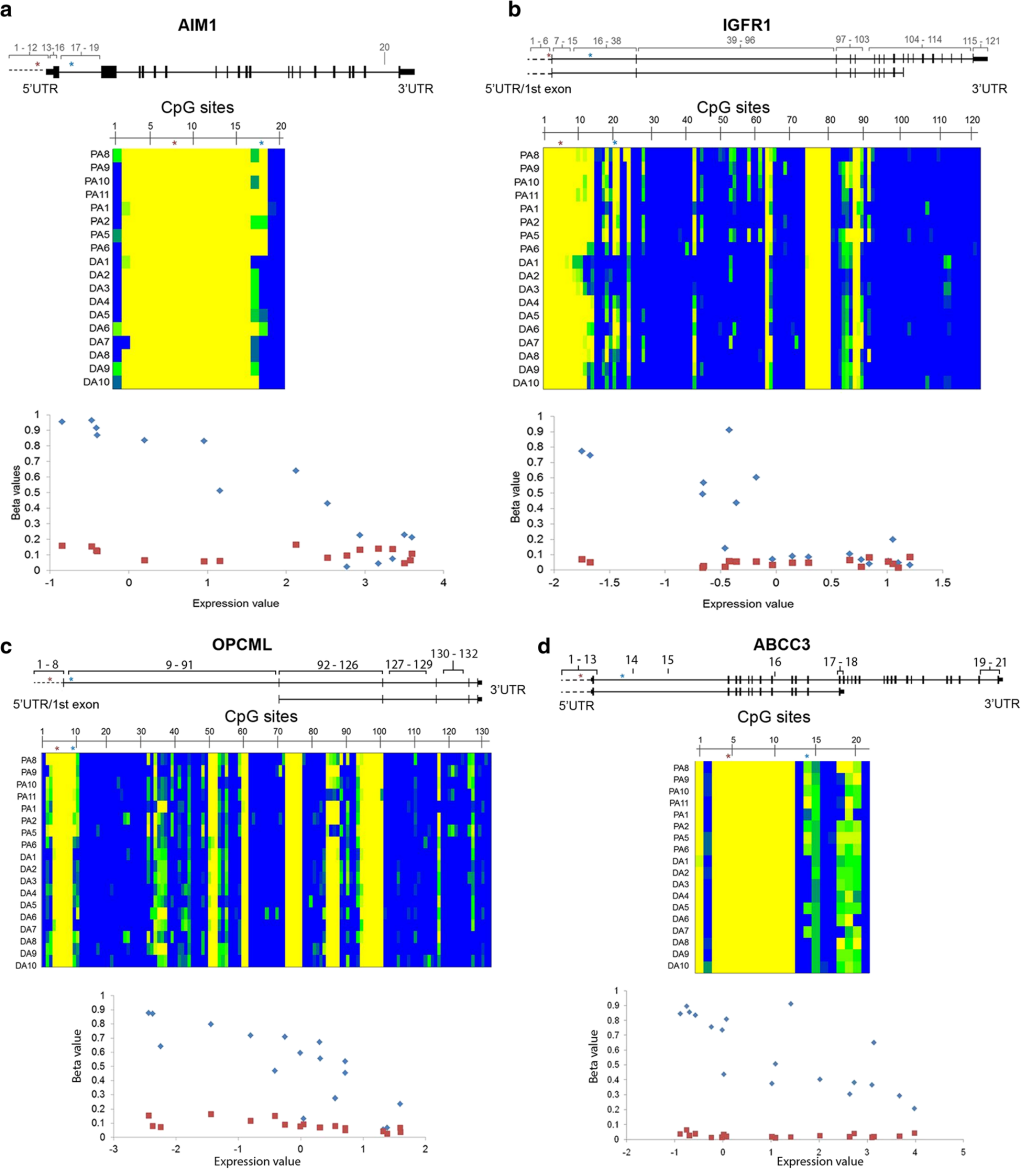
Intragenic CpG sites showing negative correlation with gene expression when promoter region is hypomethylated. The 6 genes all have differentially methylated CpG within the first intron, which show greater negative correlation (with FDR corrected *p*-values <0.05) than a CpG site within the TSS200. **a** *AIM1* (218568_at, cg14426428 *r* = -0.92, *p*-value <6.04E-0.6, cg18405900 *r* = -0.41, *p*-value <0.52). **b** *IGF1R* (203627_at, cg26577252 *r* = -0.81, *p*-value <0.00008, cg20479870 *r* = 0.08, *p*-value <0.94). **c** *OPCML* (206215_at, cg14872762 *r* = -0.82, *p*-value <0.0007, cg23236270 *r* = -0.66, *p*-value <0.122). **d** *ABCC3* (209641_at, cg25928474 *r* = -0.77, *p*-value <0.002, cg20633883 *r* = -0.34, *p*-value <0.68). (Affymetrix expression probe, differential CpG probe, TSS200 probe, correlation coefficients for each CpG probe against expression probes (r-value) and significance (FDR corrected B-H *p*-values) are shown, respectively. The gene region is shown with CpG probes, for the whole gene, represented by numbers. The heatmaps show the beta values for the CpG sites for 8 pilocytic and 10 diffuse astrocytomas that have expression data (beta values yellow, unmethylated 0 – blue, methylated 1). The CpGs are numbered as shown in the gene region. Blue asterisk - enhancer CpG, red asterisk – TSS200 CpG. In the correlation graphs promoter (TSS200) probe values are shown as red squares and the differential CpG values are shown as blue diamonds

Correlation was then assessed between gene expression and methylation for the 70 differentially methylated CpG sites in the 56 genes (Additional file 13: Table S12). When the CpG site was located within the gene body or intergenic regions, correlation was also examined for a CpG site within the promoter region. Significant correlation was identified in 36 genes for 47 CpG sites, 12 of which were located in promoter regions and 35 were in the gene body or in the intergenic regions (Fig. 3). Examples of the correlations are shown in Fig. 4. The functions of the 36 genes are presented in Table 1 and Additional file 14: Table S13. The genes identified are involved in cell morphology, cell signalling and some are downstream targets of the MAPK pathway.

**Table 1:** Functions of genes which show a significant correlation between DNA methylation and gene expression. The majority of genes were hypomethylated and up-regulated in pilocytic astrocytomas compared to diffuse astrocytomas, except the three genes highlighted in bold

MAPK pathway and downstream targets: *DUSP5* *FOS* *MIR21* *CDK6* Tumour suppressors: *AIM1* *OPCML* ***MCC*** ***IRX1*** Multidrug resistance: *ABCC3* Inflammation: *SOCS3* *IL6ST* *CD59* Other: *FAM89A* *FAM167A* *PRSS23* *WSCD1* *STAMBPL1* *TMEM169*	Receptors and channels: *HRH1* *LDLRAD3* *RARRES1* *IGF1R* *GRIA4* *CHRM3* *TRPM8* *KCND2* *GJA1* Cell morphology, motility and interactions: *MDGA2* *TM4SF1* *TMEM132C* *IGSF9B* ***KIRREL3*** *LEPREL1* *TMEM49* *COTL1* *PTPRE*

### Identification of AP-1 binding sites at the differentially methylated CpG sites and up-regulation of AP-1 targets in pilocytic astrocytomas

**Fig. 5:**
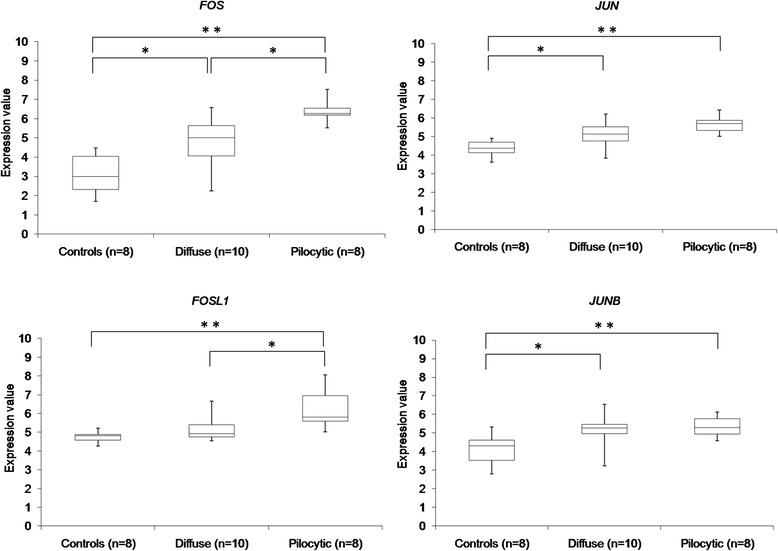
Expression of the AP-1 factors of the FOS and JUN families. Expression levels of AP-1 factors in normal brain controls (Foetal controls – diencephalon, cerebellum, cerebral cortex; Adult controls – diencephalon, medulla, thalamus, cerebellum, cerebral cortex), diffuse astrocytomas and pilocytic astrocytomas. The normalized expression values are log2 transformed. Affymetrix probe identifiers are shown (** *p*-value <0.01, * *p*-value <0.05, Mann-Whitney U-test)

Hypomethylation at enhancer regions is reported to be tissue-specific and may reflect the presence of specific transcription factors at these sites [16, 50]. We therefore examined the genomic regions around the 315 differentially methylated CpG sites to identify transcription factor binding motifs [15]. The most frequently identified transcription factor binding sites were AP-1 and AML1α. Both AP-1 and AML1α were up-regulated in pilocytic astrocytomas (Additional file 15: Table S14). The AP-1 transcription factors *FOS* and *FOSL1* were found to be significantly up-regulated in the pilocytic astrocytomas compared to diffuse astrocytomas and normal brain controls (Fig. 5; Additional file 3: Figure S12). Further analysis also revealed that a subset of published and predicted targets of AP-1 is up-regulated in pilocytic astrocytomas, including the cyclin D1 gene *CCND1* (Additional file 16: Table S15).

**Fig. 6:**
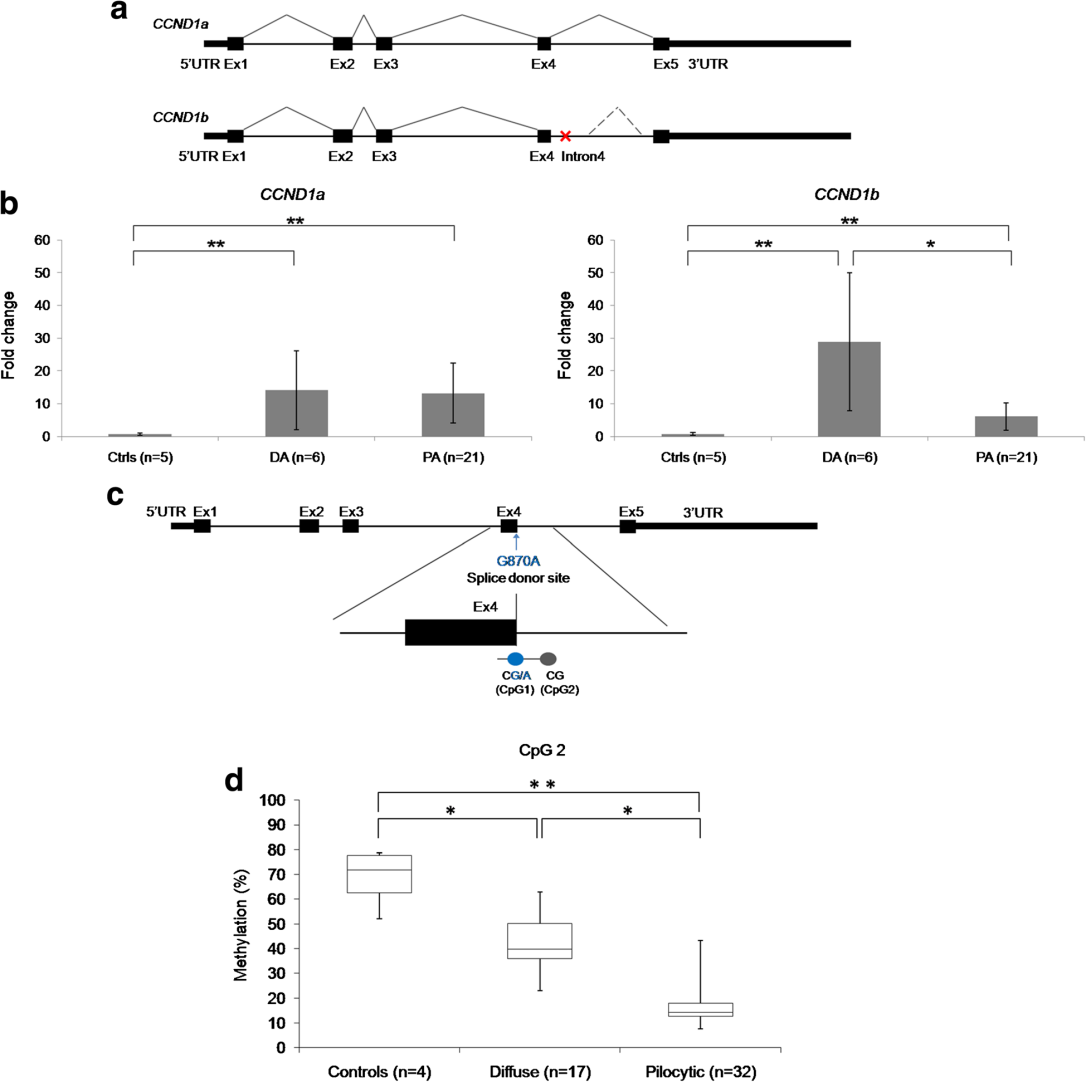
Analysis of AP-1 transcription factors and the AP-1 target gene *CCND1*. **a** Diagram showing the *CCND1a* transcript and the oncogenic splice variant *CCND1b.* Alternative splicing at the exon4-intron 4 boundary leads to the *CCND1b* transcript that contains intron 4, with loss of the post-transcriptional regulatory domain of exon 5 and 3’UTR. A stop codon (shown by the red cross) within the intron leads to truncated cyclin D1 protein. **b** Expression levels of *CCND1a* and *CCND1b* in controls, pilocytic and diffuse astrocytomas (Error bars show the standard deviation for the average beta value for each tumour group). **c** Diagram of *CCND,* showing the location of SNP rs9344 (G870A) within a CpG at the splice site (CpG1) and its neighbouring CpG site within the intron (CpG2). **d** CpG2, the closest intronic CpG site to the SNP, shows lower methylation levels in the low-grade astrocytomas compared to normal brain controls (differential change >30 %; Mann-Whitney U-test- ** *p*-value <0.01, * *p*-value <0.05)

*CCND1* regulates the cell cycle, and encodes two splice variants, *CCND1a* and an oncogenic form, *CCND1b* (Fig. 6a). While expression of both *CCND1a,* and *CCND1b* was higher in tumours than normal brain controls, diffuse astrocytomas expressed far more oncogenic *CCND1b* than pilocytic astrocytomas (Fig. 6b). Interestingly, the SNP rs9344 (G870A), which is located within a CpG site at the splice site at exon 4 - intron 4, is abolished in the presence of the A-allele and has been reported to be associated with expression of *CCND1b* [7, 48] (Fig. 6c). We then examined DNA methylation at the splice site of the exon 4 – intron 4 boundary, which was not covered by the Infinium HumanMethylation450 BeadChip. A 372 bp region covering all of exon 4 and 224 bp of intron 4, including the SNP, was Sanger-sequenced to identify the qualitative methylation status of the 11 CpG sites located within this region (Additional file 3: Figure S13). Differential methylation was identified at the adjacent CpG site to the SNP showing hypomethylation in pilocytic astrocytomas. This finding was confirmed by pyrosequencing. Although differential methylation was identified for these tumours, no significant correlation was identified between methylation and *CCND1b* expression (Additional file 3: Figure S13). Additionally, no correlation was identified between allele status of the SNP and *CCND1b* expression (Additional file 3: Figure S13).

### Comparison of low-grade astrocytomas with normal brain tissue from tumour matched locations

We then examined DNA methylation profiles for infratentorial and supratentorial pilocytic astrocytomas and diffuse astrocytomas, as separate groups, with control tissue appropriate to their respective locations. Published control profiles for cerebellum and cerebral cortex were included [12, 27]. Comparison of infratentorial pilocytic astrocytomas with all cerebellar controls identified 11,671 differentially methylated CpG sites (FDR-corrected *p*-value <0.05, Δβ > 0.3) (Additional file 17: Table S16; Additional file 4: Table S3, Panel B). The large number of differentially methylated CpG sites could be due to normal cerebellum having a greater neuronal component (3:1 neuronal to non-neuronal) than the tumours, which would contain mainly glial cells. Of the 11,671 differentially methylated sites, 8,108 were associated with 3,925 genes (Additional file 17: Table S16). The distribution of these differentially methylated CpG sites across gene regions is shown in Additional file 3: Figure S14. Analysis of gene expression profiles in these tumours showed that 146 (3.3 %) of the genes had ≥2 fold differences compared to all the cerebellum controls as a single group. Ingenuity Pathway Analysis revealed that the top five pathways associated with the 146 deregulated genes are *cellular movement, cancer, amino acid metabolism, cellular growth* and *proliferation,* with the functions including *neuritogenesis* and *neuron development* (Additional file 18: Table S17). This was in agreement with previous findings [27].

Comparison of supratentorial pilocytic astrocytomas and diffuse astrocytomas with all cerebral cortex controls identified 382 and 58 CpG sites (43 sites in common), respectively, that were differentially methylated (Additional file 4: Table S3, Panel C and D; Additional file 19: Table S18 and Additional file 20: Table S19). Comparison of supratentorial pilocytic astrocytomas and diffuse astrocytomas with glial and neuronal components of cerebral cortex [12] showed that the tumours were more closely related to the glial component, as expected (Additional file 19: Table S18 and Additional file 20: Table S19, Additional file 4: Table S3, Panel E; Additional file 9: Table S8). Hierarchical clustering of the 397 CpG sites clustered all the controls, with two major branches for the majority of the pilocytic astrocytomas and diffuse astrocytomas (Additional file 3: Figure S15). Of the genes that have differential methylation, expression of the down-regulated genes in the pilocytic astrocytomas are involved in *neuronal differentiation* and *brain function*, with the up-regulated genes involved in the *inflammatory response*, *apoptosis, metabolic processes* and *MAPK pathway* (Additional file 19: Table S18).

Finally, comparison of infratentorial and supratentorial pilocytic astrocytomas identified only 72 differentially methylated sites, most of which were relatively hypomethylated in infratentorial tumours **(**Additional file 4: Table S3, Panel F; Additional file 21: Table S20). All 72 CpG sites were also hypomethylated in the infratentorial pilocytic astrocytomas compared to the diffuse astrocytomas. CpG sites included in this group were associated with 28 genes, including *NR2E1* and *EMX2OS*, which had 9 and 3 differentially methylated sites, respectively (Additional file 3: Figure S16 and Additional file 3: Figure S17). A further 21 genes were associated with the intergenic CpG sites, identifying 49 genes as being differentially methylated. From the 49 genes, six genes showed differential expression between the two tumour groups (*SHH* and *NR2E1* up-regulated in supratentorial astrocytomas; *IRX2, IRX1, HS3ST1* and *LNX1* up-regulated in infratentorial astrocytomas).

## Discussion

We have found that pilocytic astrocytomas have a hypomethylation signature that is independent of tumour location and is absent in other types of paediatric and adult gliomas and normal brain tissue. A subset of the CpG sites is located at intragenic distal enhancer regions of low CpG density, as identified in other malignancies including medulloblastoma [17, 25, 54]. Furthermore, tissue-specific hypomethylation has been found at distal regulatory regions of genes [16]. In a subset of cancer-related genes that are associated with the distinctive signature in pilocytic astrocytomas, the affected sites within the gene body showed greater correlation with expression than the promoter region. Additionally, we identified consensus AP-1 binding sites located at a subset of the affected enhancers in pilocytic astrocytomas and an up-regulation of AP-1 factors and target genes in pilocytic astrocytomas compared to diffuse astrocytomas and normal brain. A high proportion of consensus AP-1 binding sites are reported to be located within introns and intergenic regions, but their function at these locations is not clear [29].

On further examination of the AP-1 target gene *CCND1,* we identified up-regulation of *CCND1a* in both pilocytic and diffuse astrocytomas compared to normal brain control. Interestingly, a truncated transcript, *CCND1b* has been shown to have oncogenic effects by transforming NIH-3 T3 cells [34, 48] and is expressed in other cancers [7]. *CCND1b* was expressed at higher levels in diffuse astrocytomas compared to pilocytic astrocytomas. In the low-grade astrocytomas, expression of *CCND1b* was not linked to SNP status at the splice site or DNA methylation at this exon-intron boundary. High expression of the oncogenic variant *CCND1b* may therefore be critical for tumorigenesis in diffuse astrocytomas, and suggests that defective splicing mechanisms may be present in these tumours, as shown for other malignancies [38, 64].

Previous studies have shown that the cerebellum contains more hypomethylated genes than the cerebral cortex [26, 63]. Furthermore, DNA methylation patterns are distinct in neuronal and non-neuronal cells, particularly at enhancers and non-CpG sites [24]. The differences in methylation patterns between brain regions could therefore, at least in part, reflect variation in the neuronal:non-neuronal cell ratio [12, 24]. These factors were reflected in our findings when we performed comparisons between the low-grade astrocytomas and normal brain from the tumour location.

Comparison between the infratentorial and supratentorial pilocytic astrocytomas identified 49 genes as being differentially methylated, of which 6 had been identified previously [27]. Our findings may vary due to our supratentorial tumours being located predominately in the cerebral cortex, whereas the tumours in the previous study came from varied supratentorial regions. A key gene identified was *NR2E1,* which is important in the regulation of neural stem cell expansion and gliomagenesis [31, 68]. It is one of the genes reported to distinguish infratentorial and supratentorial pilocytic astrocytomas by their expression and/or DNA methylation profiles [27, 46]. We also identified other homeobox genes *EMX2OS, MEIS1* and *PBX3* that play a role in brain development [2, 13, 44, 52, 58].

It remains to be established whether the differences that we have observed arise from the specific genetic changes in pilocytic and diffuse astrocytomas, or whether the profiles reflect the methylation pattern in the cell of origin [9, 36, 41]. Distinct outputs of the MAPK pathway have been identified in melanoma and other malignancies with *BRAF*^*V600E*^ and receptor tyrosine kinase mutations. Additionally, genetic alterations in mouse models of brain tumours often interfere with normal differentiation processes and give rise to tumours in a manner that is dependent on the tumour cell of origin, as reviewed recently [53]. Pilocytic astrocytomas in the optic pathway are believed to derive from radial glia cells that are distinct from those giving rise to cerebellar pilocytic astrocytomas [28, 57]. The identity of the tumour precursor cells in non-optic pathway pilocytic astrocytomas, however, remains elusive, even in mouse models [6]. The discovery of a unique methylation signature at a small number of sites may facilitate the identification of tumour precursor cells and in time enable the very earliest stages of tumorigenesis to be better understood.

## Conclusion

In summary, pilocytic astrocytomas contain a hypomethylation signature characterised by CpG sites which are located predominantly in annotated enhancers. This signature is specific to pilocytic astrocytomas and is not present in diffuse astrocytomas, other brain tumours or normal brain tissue. The AP-1 transcription factor complex, activated by the MAPK pathway, is predicted to bind at a number of these CpG sites, and FOS transcription factors are up-regulated in pilocytic astrocytomas. Our findings highlight epigenetic differences between pilocytic and diffuse astrocytoma, in addition to the well-documented genomic alterations.

## Additional files

Additional file 1: Table S1. Study tumour set, validation tumour set and controls. Clinical features of patients, tumour pathology and genetic changes are shown. Samples that were included in the whole genome sequencing (WGS) study of the Pediatric Cancer Genome Project (PCGP) are noted together with the original patient numbers [Zhang et al, Nature Genetics 45(6):602-614, 2013]. (XLSX 406 kb) Additional file 2: Table S2. Peak-corrected Infinium 450K methylation beta values for the test tumour set. Beta values are the ratios between the unmethylated (0) and methylated (1) signal intensities for the individual probes (ordered by Illumina probe ID). Normal brain controls: Foetal cerebellum, Foetal brain, Foetal frontal lobe, adult brain, human neural stem cells. Supratentorial pilocytic astrocytomas: PA1-PA6. Infratentorial pilocytic astrocytomas: PA7-PA17. Diffuse astrocytomas: DA1-DA10. (XLSX 173351 kb) Additional file 3: Supplementary Methods; Legends to Supplementary Tables; Supplementary Figures. (PDF 1904 kb) Additional file 4: Table S3. Overview of comparisons performed between pilocytic astrocytomas and diffuse astrocytomas, study controls, and normal brain tissue. Differentially methylated CpG sites (δβ ≥0.3, FDR-corrected *p*-value <0.05) were identified by MethLAB analysis. (PDF 104 kb) Additional file 5: Table S4. Normalized gene expression analysis for selected test tumours and controls. Expression analysis was performed using Affymetrix Human U133_plus2 arrays. Log-transformed expression values are shown for 18 tumour samples (10 diffuse astrocytomas and 8 pilocytic astrocytomas), and foetal and adult normal brain controls (foetal and adult diencephalon, cerebellum and cerebral cortex, adult medulla and thalamus). Affymetrix annotation is shown. Probes are ordered alphabetically according to the gene symbol. (XLSX 19307 kb) Additional file 6: Table S5. Pyrosequencing assays. Primers used for pyrosequencing (XLSX 72 kb) Additional file 7: Table S6. Three-way comparison of the test tumour set of pilocytic and diffuse astrocytomas with normal brain control tissue. 993 CpG probes are shown where the difference between the average beta values of pilocytic and diffuse astrocytomas, or between either of these and the control samples is equal to or greater than 0.3 (δβ ≥ 0.3, Benjamini-Hochberg corrected *p*-value <0.05), assessed by MethLAB. Illumina annotation is included. (XLSX 436 kb) Additional file 8: Table S7. Ingenuity Pathway Analysis (IPA) for genes associated with 993 differentially methylated sites identified in the three-way comparison of the test tumour set. IPA analysis shows significant biological and disease categories including functional annotation (*p*-value <0.05), for 537 genes sorted by *p*-value. (XLSX 89 kb) Additional file 9: Table S8. CpG sites which are present or absent from the hypomethylation signature comparisons between the test tumour set, published normal brain controls and published tumour sets. The 311 CpG sites were compared to published normal controls and tumour sets, and the data represented as heatmaps in Additional file 3: Figures S13-S16. Some of the CpG sites were not represented in the comparison datasets; therefore the lists of present CpG sites are shown. The 311 CpG sites are ordered from low to high methylation in the pilocytic astrocytomas. Normal brain controls (1_Cerebellum [Lambert et al, Acta Neuropathologica 126:291-301, 2013]; Cerebral cortex [Guintivano et al, Epigenetics 8:290-302, 2013]); paediatric low-grade astrocytomas (2_validation tumour set 2; 3_[Lambert et al, Acta Neuropathologica 126:291-301, 2013]); adult low-grade astrocytomas (4_TCGA); paediatric and adult high-grade astrocytomas (5_[Sturm et al, Cancer Cell 22:425-437, 2012]), medulloblastomas (6_ [Schwalbe et al, Acta Neuropathologica 126:943-946, 2013]; Ependymomas (7_GSE45353 [Mack et al, Nature 506:445-450, 2014]) and foetal brain during development (8_GSE58885 [Spiers et al, Genome Research 25:338-352, 2015]). (XLSX 320 kb) Additional file 10: Table S9. Comparison of pilocytic astrocytomas with diffuse astrocytomas in the test tumour set. 315 CpG sites are shown where δβ ≥ 0.3 (Benjamini-Hochberg corrected *p*-value <0.05), assessed by MethLAB. Illumina annotation is included. Rows highlighted in grey (42/315 CpG sites) were absent in the 993 differentially methylated CpG sites between tumours and normal brain control. Ordered by chromosomal location. (XLSX 205 kb) Additional file 11: Table S10. Ingenuity Pathway Analysis (IPA) for genes associated with 315 differentially methylated sites in the test pilocytic and diffuse astrocytomas. 1_ The 185 genes that were directly associated with 217/315 CpG sites. Enhancers (grey) and CpG probes that are present within more than one gene (#) are highlighted. 2_ IPA analysis shows significant biological and disease categories which includes their functional annotations (*p*-value <0.05), sorted by *p*-value. (XLSX 116 kb) Additional file 12: Table S11. Normalised log2 expression values for the genes associated with the 315 CpG sites. The δβ values between the pilocytic and diffuse astrocytoma groups were obtained from the grouped averaged beta values. Genes with a differential gene expression ≥2 fold are highlighted in grey. Sorted alphabetically by gene symbol. (XLSX 370 kb) Additional file 13: Table S12. Comparison of methylation and expression for genes associated with 315 differentially methylated sites in pilocytic and diffuse astrocytomas. Correlation coefficients are shown for differentially methylated CpG sites that show a difference of ≥2-fold in expression of the associated gene. Expression probes were only assessed if they were located within the mRNA transcript. A) CpG sites located within the promoter of the gene. B) CpG sites located within the gene body, 3’UTR and intergenic regions. As the CpG is not within a promoter, correlation was also calculated for a CpG site within the promoter (where possible within the TSS200; brown panel). For each correlation *p*-values and Benjamini-Hochberg corrected *p*-values are shown. The table is ordered by FDR-corrected *p*-values (sites with a *p*-value <0.05 are shown in blue). Positive correlation is highlighted in yellow. Up-regulated genes in diffuse astrocytomas are highlighted in green. When the expression probe was located in an exon, the probe for the full transcript was used for the correlation (indicated by the asterisk). (XLSX 85 kb) Additional file 14: Table S13. Functions of genes which show a significant correlation between DNA methylation and gene expression. The majority of genes were hypomethylated in pilocytic astrocytomas except the genes highlighted in bold, which were hypermethylated in the pilocytic astrocytomas. (PDF 158 kb) Additional file 15: Table S14. Predicted transcription factor binding sites for the 315 differentially methylated signature in low-grade astrocytomas. Table to show TRANSFAC predicted transcription factor binding sites within a 200 bp region (100 bp either side of the CpG site detected by the Illumina probe). Illumina annotation highlighted in grey and transcription factor binding predictions plus sequence highlighted in blue. List of the top predicted transcription factors and gene expression levels of the top predicted transcription factors for the low-grade astrocytomas are also shown. (XLSX 215 kb) Additional file 16: Table S15. Differential expression of AP-1 target genes in pilocytic astrocytomas compared to normal brain controls and diffuse astrocytomas. 1_ List of published AP-1 target genes [Eferl and Wagner, Nature Rev Cancer 3(11):853-868, 2003; Li et al, J Genet Genomics 38(6):235-242, 2011] and predicted AP-1 target genes identified in the 315 differentially methylated sites. 2_ Table showing AP-1 target genes that are differentially expressed in pilocytic astrocytomas compared to normal brain controls and diffuse astrocytomas. For each gene, methylation and expression status, plus the normalized log2 transformed expression values for each sample are included. The data is ordered by correlation with FOS expression (positive to negative correlation). The colour coordination shows which genes were taken from the three lists. (XLSX 101 kb) Additional file 17: Table S16. The differentially methylated CpG sites within de-regulated genes from the comparison between infratentorial pilocytic astrocytomas and normal cerebellum controls. 1_ Features of 11, 671 probes that are significantly differentially methylated in infratentorial pilocytic astrocytomas compared to published foetal and adult normal cerebellum datasets (averaged beta values with a difference of δβ ≥ 0.3, Benjamini-Hochberg corrected *p*-value <0.05 [Lambert et al, Acta Neuropathologica 126:291-301, 2013]). 2_ Normalized log2 transformed expression analysis identifies differentially expressed genes between infratentorial pilocytic astrocytomas (*n* = 4) compared to normal cerebellum (*n* = 2, foetal and adult cerebellum). Genes that show fold change >2 are highlighted. 3_ List of genes that have differential methylation (δβ ≥ 0.3) and differential gene expression (fold change >2). (XLSX 4414 kb) Additional file 18: Table S17. Ingenuity Pathway Analysis (IPA) of genes associated with differentially methylated sites in infratentorial pilocytic astrocytomas and normal cerebellum. IPA analysis of the top 20 significant biological and disease categories including their functional annotation (*p*-value <0.05) identified for 161 genes that had ≤2 fold change in gene expression and δβ ≥ 0.3, sorted by *p*-value. (XLSX 71 kb) Additional file 19: Table S18. Differentially methylated CpG sites identified between supratentorial pilocytic astrocytomas and cerebral cortex controls. 1_ MethLAB analysis identified 90,249 CpG sites that are significantly differentially methylated in supratentorial pilocytic astrocytomas, diffuse astrocytomas, glial component of the cerebral cortex, neuronal component of the cerebral cortex, adult cerebral cortex and foetal cerebral cortex (Benjamini-Hochberg corrected *p*-value <0.05). The adult cerebral cortex controls (including the glial and neuronal components) were taken from [Guintivano et al, Epigenetics 8:290-302, 2013]. The number of differentially methylated sites (averaged beta values with a difference of δβ ≥ 0.3, Benjamini-Hochberg corrected *p*-value <0.05) identified in tumour and all controls was 382 CpG sites for supratentorial pilocytic astrocytomas. 2_ CpG sites hypermethylated and 3_ hypomethylated in pilocytic astrocytomas compared to controls. 4_ Expression analysis for the genes that show differentially methylated CpG sites. Highlighted genes show ≤ 2 fold change in expression compared to controls. (XLSX 37842 kb) Additional file 20: Table S19. Differentially methylated CpG sites identified between diffuse astrocytomas and cerebral cortex controls. 1_ MethLAB analysis identified 90,249 CpG sites that are significantly differentially methylated in supratentorial pilocytic astrocytomas, diffuse astrocytomas, glial component of the cerebral cortex, neuronal component of the cerebral cortex, adult cerebral cortex and foetal cerebral cortex (Benjamini-Hochberg corrected *p*-value <0.05). The adult cerebral cortex controls (including the glial and neuronal components) were taken from [Guintivano et al, Epigenetics 8:290-302, 2013]. The number of differentially methylated sites (averaged beta values with a difference of δβ ≥ 0.3, Benjamini-Hochberg corrected *p*-value <0.05) identified in tumour and all controls was 58 CpG sites for the diffuse astrocytomas. 2_ CpG sites hypermethylated and 3_ hypomethylated in diffuse astrocytomas compared to controls. 4_ Expression analysis for the genes that show differentially methylated CpG sites. Highlighted genes show ≤ 2 fold change in expression compared to controls. (XLSX 37720 kb) Additional file 21: Table S20. Differentially methylated CpG sites in infratentorial pilocytic astrocytomas, supratentorial astrocytomas and diffuse astrocytomas. 1_ MethLAB analysis identified 676 CpG sites that are significantly differentially methylated in infratentorial pilocytic, supratentorial pilocytic and diffuse astrocytomas (Benjamini-Hochberg corrected *p*-value <0.05). 2_ The 72 differentially methylated CpG sites in supratentorial and infratentorial pilocytic astrocytomas. 3_ The 393 differentially methylated CpG sites for infratentorial pilocytic astrocytomas and diffuse astrocytomas (averaged beta values with δβ ≥ 0.3, Benjamini-Hochberg corrected *p*-value <0.05). 4_ Genes that show differentially methylated CpG sites and expression between supratentorial and infratentorial pilocytic astrocytomas (genes with fold change ≥ 2 highlighted in grey). (XLSX 406 kb)
