# Supplementary material for: DNA methylation analysis of paediatric low-grade astrocytomas identifies a tumour-specific hypomethylation signature in pilocytic astrocytomas
Source: Acta Neuropathol Commun. 2016 May 27;4:54. doi: 10.1186/s40478-016-0323-6 (PMC4882864; doi:10.1186/s40478-016-0323-6)
Supplement: Additional file 3: — Supplementary Methods; Legends to Supplementary Tables; Supplementary Figures. (PDF 1904 kb) [file 40478_2016_323_MOESM3_ESM.pdf]

Additional file 3: Supplementary Information for:

DNA methylation analysis of paediatric low-grade astrocytomas identifies a tumour-specific signature at a set of enhancers in pilocytic astrocytomas

Jennie N. Jeyapalan[1], Gabriel T. Doctor[1], Tania A. Jones[1], Samuel N. Alberman[1], Alexander Tep[1], Chirag M. Haria[1], Ed C. Schwalbe[2,3], Isabel C. F. Morley[1], Alfred A. Hill[1], Magdalena LeCain[1], Diego Ottaviani[1], Steven C. Clifford[2], Ibrahim Qaddoumi[4], Ruth G. Tatevossian[5], David W. Ellison[5]* and Denise Sheer[1]*

[1]Blizard Institute, Barts and The London School of Medicine and Dentistry, Queen Mary University of London, 4 Newark Street, London, UK. [2]Northern Institute for Cancer Research, Newcastle University, Newcastle upon Tyne, UK. [3]Department of Applied Sciences, Northumbria University, Newcastle upon Tyne, UK. [4]Department of Oncology, St Jude Children's Research Hospital, Memphis, TN, USA. [5]Department of Pathology, St Jude Children's Research Hospital, Memphis, TN, USA.

***Correspondence to:**

D.W. Ellison
Department of Pathology,
St Jude Children's Research Hospital,
Memphis, TN 38105-3678,
USA
Phone: +1 901 595-5438
Fax: +1 901 595-3100
Email: david.ellison@stjude.org

D. Sheer
Blizard Institute
4 Newark Street,
London, E1 2AT,
UK
Tel: +44 207 882 2595
Fax: + 44 207 882 2180
Email: d.sheer@qmul.ac.uk

Contents:

Supplementary Methods	p.2
Legends for Supplementary Tables	p.4
Supplementary Figures	p.9
References	p.35

SUPPLEMENTARY METHODS

Differential methylation analysis

Differential methylation analysis was performed using the MethLAB R-based programme [4] using R (version 2.15.0). The program enables us to identify significantly differentially methylated CpGs from the corrected beta values. It utilises a t-statistic model, with linear regression and FDR correction. For the analysis, the beta values were converted to M values.

M-value = log2 [Beta value/1-Beta value]

The linear model with the factor of interest (tumour type – pilocytic, diffuse, control) was computed, with other varying factors; bead chip number (1-5), BRAF status (fusion, V600E mutation, WT), sample location (infratentorial/supratentorial), age group (fetal/HNSC, <3 years, >=3 years, >16 years) and sex (male, female) included in the model. A class covariance and FDR correction (Benjamini-Hochberg) were performed. Files entered into the program are the corrected β-values for each sample (Illumina probe ID and β-values excluding probes on the Chr X and Y), a phenotype file (includes sample group (pilocytic, diffuse, normal brain controls), bead chip, BRAF status, sample location, age and sex) and the 450K annotation file. Comparisons performed are shown below (statistically significant CpG sites had FDR-corrected *p*-value <0.05). The linear model is the linear relationship between dependent variable and independent variables (n). In this analysis the dependent variable is the β-value for each probe and the independent variables are the phenotypic factors such as tumour type. To identify tumour-specific changes the other independent variables are taken into account. For identification of key differentially methylated CpG sites it has been shown that the recommended sensitivity for differential analysis using the Illumina bead chip technology is a delta beta value >0.2 [1]. For our analysis, differentially methylated CpG sites of interest had a differential change of ≥0.3 with FDR-corrected *p*-value <0.05. Results of the comparisons are shown in Supplementary Table 3.

MethLAB comparison analyses-

1) Pilocytic astrocytoma (n=17) v diffuse astrocytoma (n=10) v controls (n=5) - Mutation analysis

The independent variable was firstly *BRAF* fusion status (*BRAF* fusion, n=12; No *BRAF* fusion, n= 20) and then *BRAF* status (*BRAF* fusion and $BRAF^{V600E}$, n=17; WT BRAF, n=15). The co-variables included are bead chip, sample type, sample location, sex and age. Both analyses identified no significant CpG sites.

2) Pilocytic astrocytoma (n=17) v diffuse astrocytoma (n=10) v controls (n=5) – Tumour type analysis

The independent variable was sample type (pilocytic, diffuse or control). The co-variables included are bead chip, sample location, sex and age. Analysis identified 10,039 CpG sites that were unique to each of the 3 groups (FDR-corrected *p*-values <0.05).

3) Infratentorial pilocytic astrocytomas (n=11) v foetal cerebellum (n=6) v adult cerebellum (n=2)

Normal cerebellum controls were taken from GSE44684 [5]. Due to large number of statistically significant sites

that were computed between the tumours and controls, MethLAB analysis was not able to be performed on the full dataset. Therefore differential changes of >0.3 between the averaged sample groups were firstly calculated. The independent variable was sample type (pilocytic or foetal cerebellum or adult cerebellum). Analysis identified 11,671 CpG sites unique to the 3 groups (FDR-corrected *p*-values <0.05).

4) Supratentorial pilocytic astrocytomas (n=6) v diffuse astrocytomas (n=10) v each normal cerebral cortex group (foetal n=1, adult n=2, glial component n=4, neuronal component n=4)

Normal cerebral cortex controls for the adult cortex were taken from GSE41826 [3]. The independent variable was sample type (pilocytic, diffuse, foetal cortex, adult cortex, glial and neuronal components). The co-variables included are bead chip and sex. Analysis identified 63,065 CpG sites that were unique to each of the 3 groups (FDR-corrected *p*-values <0.05).

LEGENDS FOR SUPPLEMENTARY TABLES

Additional file 1: Table S1

Study tumour set, validation tumour set and controls

Clinical features of patients, tumour pathology and genetic changes are shown. Samples that were included in the whole genome sequencing (WGS) study of the Pediatric Cancer Genome Project (PCGP) are noted together with the original patient numbers [Zhang et al, Nature Genetics 45(6):602-614, 2013].

Additional file 2: Table S2

Peak-corrected Infinium 450K methylation beta values for the test tumour set

Beta values are the ratios between the unmethylated (0) and methylated (1) signal intensities for the individual probes (ordered by Illumina probe ID). Normal brain controls: Foetal cerebellum, Foetal brain, Foetal frontal lobe, adult brain, human neural stem cells. Supratentorial pilocytic astrocytomas: PA1-PA6. Infratentorial pilocytic astrocytomas: PA7-PA17. Diffuse astrocytomas: DA1-DA10.

Additional file 4: Table S3

Overview of comparisons performed between pilocytic astrocytomas and diffuse astrocytomas, study controls, and normal brain tissue

Differentially methylated CpG sites ($\delta\beta \geq 0.3$, FDR-corrected p-value <0.05) were identified by MethLAB analysis.

Additional file 5: Table S4

Normalized gene expression analysis for selected test tumours and controls

Expression analysis was performed using Affymetrix Human U133_plus2 arrays. Log-transformed expression values are shown for 18 tumour samples (10 diffuse astrocytomas and 8 pilocytic astrocytomas), and foetal and adult normal brain controls (foetal and adult diencephalon, cerebellum and cerebral cortex, adult medulla and thalamus). Affymetrix annotation is shown. Probes are ordered alphabetically according to the gene symbol.

Additional file 6: Table S5

Pyrosequencing assays

Primers used for pyrosequencing

Additional file 7: Table S6

Three-way comparison of the test tumour set of pilocytic and diffuse astrocytomas with normal brain control tissue

993 CpG probes are shown where the difference between the average beta values of pilocytic and diffuse astrocytomas, or between either of these and the control samples is equal to or greater than 0.3 ($\delta\beta \geq 0.3$, Benjamini-Hochberg corrected p-value <0.05), assessed by MethLAB. Illumina annotation is included.

Additional file 8: Table S7

Ingenuity Pathway Analysis (IPA) for genes associated with 993 differentially methylated sites identified in the three-way comparison of the test tumour set

IPA analysis shows significant biological and disease categories including functional annotation (p-value <0.05), for 537 genes sorted by p-value.

Additional file 9: Table S8

CpG sites which are present or absent from the hypomethylation signature comparisons between the test tumour set, published normal brain controls and published tumour sets

The 311 CpG sites were compared to published normal controls and tumour sets, and the data represented as heatmaps in Supplementary Figures 13 - 16. Some of the CpG sites were not represented in the comparison datasets; therefore the lists of present CpG sites are shown. The 311 CpG sites are ordered from low to high methylation in the pilocytic astrocytomas. Normal brain controls (**1_**Cerebellum [Lambert et al, Acta Neuropathologica 126:291-301, 2013]; Cerebral cortex [Guintivano et al, Epigenetics 8:290-302, 2013]); paediatric low-grade astrocytomas (**2_**validation tumour set 2; **3_**[Lambert et al, Acta Neuropathologica 126:291-301, 2013]); adult low-grade astrocytomas (**4_**TCGA); paediatric and adult high-grade astrocytomas (**5_[**Sturm et al, Cancer Cell 22:425-437, 2012]), medulloblastomas (**6_** [Schwalbe et al, Acta Neuropathologica 126:943-946, 2013]; Ependymomas (**7_**GSE45353 [Mack et al, Nature 506:445-450, 2014]) and foetal brain during development (**8_**GSE58885 [Spiers et al, Genome Research 25:338-352, 2015]).

Additional file 10: Table S9

Comparison of pilocytic astrocytomas with diffuse astrocytomas in the test tumour set

315 CpG sites are shown where $\delta\beta \geq 0.3$ (Benjamini-Hochberg corrected p-value <0.05), assessed by MethLAB. Illumina annotation is included. Rows highlighted in grey (42/315 CpG sites) were absent in the 993 differentially methylated CpG sites between tumours and normal brain control. Ordered by chromosomal location.

Additional file 11: Table S10

Ingenuity Pathway Analysis (IPA) for genes associated with 315 differentially methylated sites in the test pilocytic and diffuse astrocytomas

1_ The 185 genes that were directly associated with 217/315 CpG sites. Enhancers (grey) and CpG probes that are present within more than one gene (#) are highlighted. **2_** IPA analysis shows significant biological and disease categories which includes their functional annotations (p-value <0.05), sorted by p-value.

Additional file 12: Table S11

Normalised log2 expression values for the genes associated with the 315 CpG sites

The $\delta\beta$ values between the pilocytic and diffuse astrocytoma groups were obtained from the grouped averaged beta values. Genes with a differential gene expression ≥2 fold are highlighted in grey. Sorted alphabetically by gene symbol.

Additional file 13: Table S12

Comparison of methylation and expression for genes associated with 315 differentially methylated sites in pilocytic and diffuse astrocytomas

Correlation coefficients are shown for differentially methylated CpG sites that show a difference of ≥2-fold in expression of the associated gene. Expression probes were only assessed if they were located within the mRNA transcript. A) CpG sites located within the promoter of the gene. B) CpG sites located within the gene body, 3'UTR and intergenic regions. As the CpG is not within a promoter, correlation was also calculated for a CpG site within the promoter (where possible within the TSS200; brown panel). For each correlation p-values and Benjamini-Hochberg corrected p-values are shown. The table is ordered by FDR-corrected p-values (sites with a p-value <0.05 are shown in blue). Positive correlation is highlighted in yellow. Up-regulated genes in diffuse astrocytomas are highlighted in green. When the expression probe was located in an exon, the probe for the full transcript was used for the correlation (indicated by the asterisk).

Additional file 14: Table S13

Functions of genes which show a significant correlation between DNA methylation and gene expression. The majority of genes were hypomethylated in pilocytic astrocytomas except the genes highlighted in bold, which were hypermethylated in the pilocytic astrocytomas.

Additional file 15: Table S14

Predicted transcription factor binding sites for the 315 differentially methylated signature in low-grade astrocytomas

Table to show TRANSFAC predicted transcription factor binding sites within a 200bp region (100bp either side of the CpG site detected by the Illumina probe). Illumina annotation highlighted in grey and transcription factor binding predictions plus sequence highlighted in blue. List of the top predicted transcription factors and gene expression levels of the top predicted transcription factors for the low-grade astrocytomas are also shown.

Additional file 16: Table S15

Differential expression of AP-1 target genes in pilocytic astrocytomas compared to normal brain controls and diffuse astrocytomas

1_ List of published AP-1 target genes [Eferl and Wagner, Nature Rev Cancer 3(11):853-868, 2003; Li et al, J Genet Genomics 38(6):235-242, 2011] and predicted AP-1 target genes identified in the 315 differentially methylated sites. **2_** Table showing AP-1 target genes that are differentially expressed in pilocytic astrocytomas compared to normal brain controls and diffuse astrocytomas. For each gene, methylation and expression status, plus the normalized log2 transformed expression values for each sample are included. The data is ordered by correlation with FOS expression (positive to negative correlation). The colour coordination shows which genes were taken from the 3 lists.

Additional file 17: Table S16

The differentially methylated CpG sites within de-regulated genes from the comparison between infratentorial pilocytic astrocytomas and normal cerebellum controls

1_ Features of 11, 671 probes that are significantly differentially methylated in infratentorial pilocytic astrocytomas compared to published foetal and adult normal cerebellum datasets (averaged beta values with a difference of $\delta\beta \geq 0.3$, Benjamini-Hochberg corrected p-value <0.05 [Lambert et al, Acta Neuropathologica 126:291-301, 2013]). **2_** Normalized log2 transformed expression analysis identifies differentially expressed genes between infratentorial pilocytic astrocytomas (n=4) compared to normal cerebellum (n=2, foetal and adult cerebellum). Genes that show fold change >2 are highlighted. **3_** List of genes that have differential methylation ($\delta\beta \geq 0.3$) and differential gene expression (fold change >2).

Additional file 18: Table S17

Ingenuity Pathway Analysis (IPA) of genes associated with differentially methylated sites in infratentorial pilocytic astrocytomas and normal cerebellum

IPA analysis of the top 20 significant biological and disease categories including their functional annotation (p-value <0.05) identified for 161 genes that had ≤2 fold change in gene expression and $\delta\beta \geq 0.3$, sorted by p-value.

Additional file 19: Table S18

Differentially methylated CpG sites identified between supratentorial pilocytic astrocytomas and cerebral cortex controls

1_ MethLAB analysis identified 90,249 CpG sites that are significantly differentially methylated in supratentorial pilocytic astrocytomas, diffuse astrocytomas, glial component of the cerebral cortex, neuronal component of the cerebral cortex, adult cerebral cortex and foetal cerebral cortex (Benjamini-Hochberg corrected p-value <0.05). The adult cerebral cortex controls (including the glial and neuronal components) were taken from [Guintivano et al, Epigenetics 8:290-302, 2013]. The number of differentially methylated sites (averaged beta values with a difference of $\delta\beta \geq 0.3$, Benjamini-Hochberg corrected p-value <0.05) identified in tumour and all controls was 382 CpG sites for supratentorial pilocytic astrocytomas. **2_** CpG sites hypermethylated and **3_** hypomethylated in pilocytic astrocytomas compared to controls. **4_** Expression analysis for the genes that show differentially methylated CpG sites. Highlighted genes show ≤ 2 fold change in expression compared to controls.

Additional file 20: Table S19

Differentially methylated CpG sites identified between diffuse astrocytomas and cerebral cortex controls

1_ MethLAB analysis identified 90,249 CpG sites that are significantly differentially methylated in supratentorial pilocytic astrocytomas, diffuse astrocytomas, glial component of the cerebral cortex, neuronal

component of the cerebral cortex, adult cerebral cortex and foetal cerebral cortex (Benjamini-Hochberg corrected p-value <0.05). The adult cerebral cortex controls (including the glial and neuronal components) were taken from [Guintivano et al, Epigenetics 8:290-302, 2013]. The number of differentially methylated sites (averaged beta values with a difference of $\delta\beta \geq 0.3$, Benjamini-Hochberg corrected p-value <0.05) identified in tumour and all controls was 58 CpG sites for the diffuse astrocytomas. **2_** CpG sites hypermethylated and **3_** hypomethylated in diffuse astrocytomas compared to controls. **4_** Expression analysis for the genes that show differentially methylated CpG sites. Highlighted genes show ≤ 2 fold change in expression compared to controls.

Additional file 21: Table S20

Differentially methylated CpG sites in infratentorial pilocytic astrocytomas, supratentorial astrocytomas and diffuse astrocytomas

1_ MethLAB analysis identified 676 CpG sites that are significantly differentially methylated in infratentorial pilocytic, supratentorial pilocytic and diffuse astrocytomas (Benjamini-Hochberg corrected p-value <0.05). **2_** The 72 differentially methylated CpG sites in supratentorial and infratentorial pilocytic astrocytomas. **3_** The 393 differentially methylated CpG sites for infratentorial pilocytic astrocytomas and diffuse astrocytomas (averaged beta values with $\delta\beta \geq 0.3$, Benjamini-Hochberg corrected p-value <0.05). **4_** Genes that show differentially methylated CpG sites and expression between supratentorial and infratentorial pilocytic astrocytomas (genes with fold change $\geq$2 highlighted in grey).

SUPPLEMENTARY FIGURES

Supplementary Figure 1. Correlation between Illumina 450K BeadChip and pyrosequencing analysis of methylation of CpG sites within 5 genes for the test tumour set

Correlation coefficients were calculated for the matching 450K (beta values) and pyrosequencing (% methylation) values at selected CpG sites (gene names and Illumina probe identifiers shown). R-values range from 0-1 with strong correlation having values close to 1. Tumours and controls tested are: pilocytic astrocytomas (PA, n=11), diffuse astrocytomas (DA, n=9) and normal brain controls (AB, Adult brain; FFL, foetal frontal lobe; FB, foetal brain; FCB, foetal cerebellum, n=4).

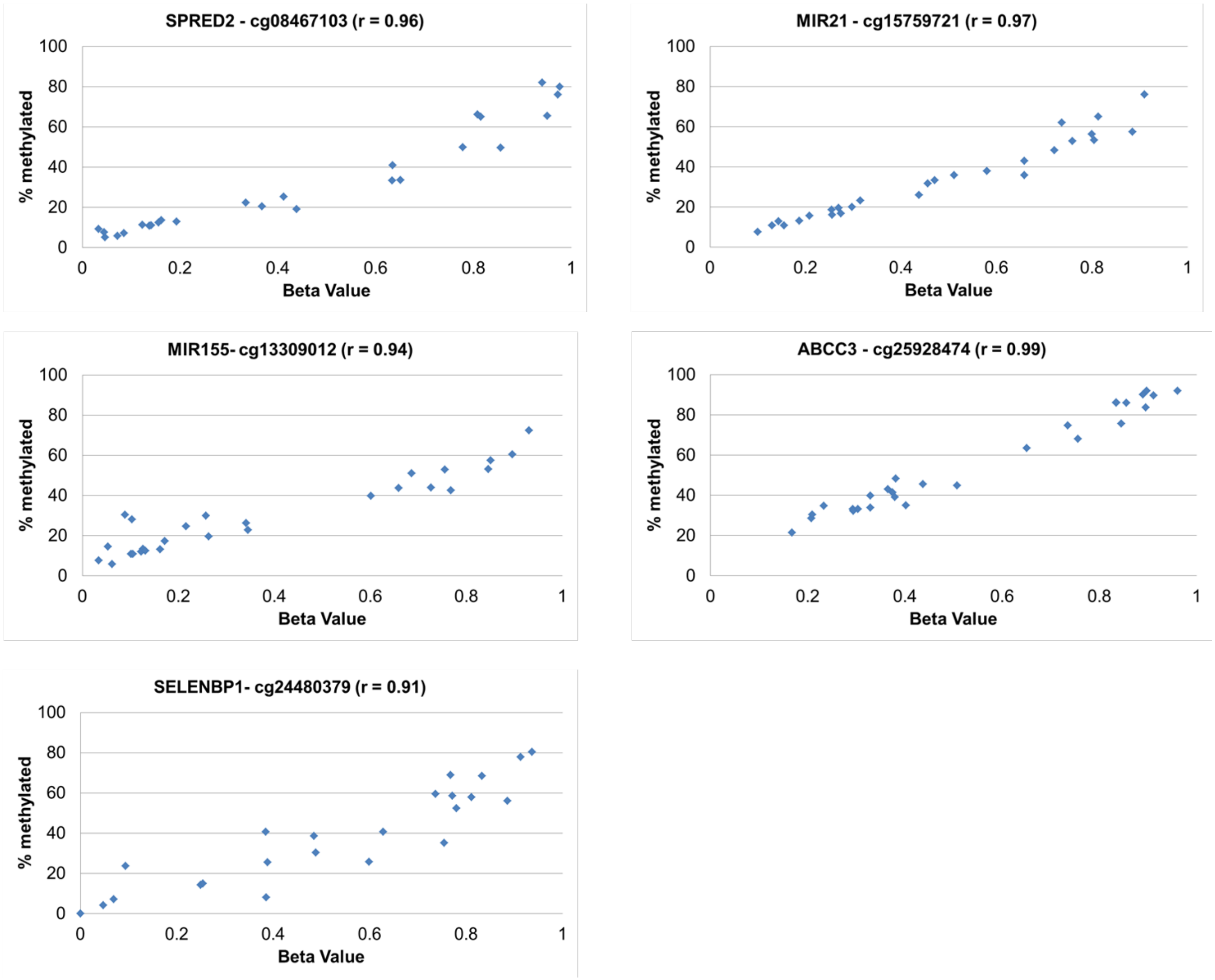

Supplementary Figure 2. Hypomethylated CpG site within the gene body of *SPRED2* is unique to pilocytic astrocytomas

(**a**) Diagram of the *SPRED2* gene highlighting differentially methylated regions (CpGs 1-3) within the gene body of the long transcript and the promoter region of short transcript. (**b**) CpG2 is hypomethylated in pilocytic astrocytomas (box plot). (**c**) Pyrosequencing validation with % methylation of CpGs 1-3, for the study and validation tumour set. Error bars present for technical replicas (two bisulphite conversions, n=2). Grey shaded samples are from test set. DA, diffuse astrocytomas; PA infra, infratentorial pilocytic astrocytomas; PA supra, supratentorial pilocytic astrocytomas; AB, adult brain; FB, foetal brain; FFL, foetal frontal lobe; FCB, foetal cerebellum.

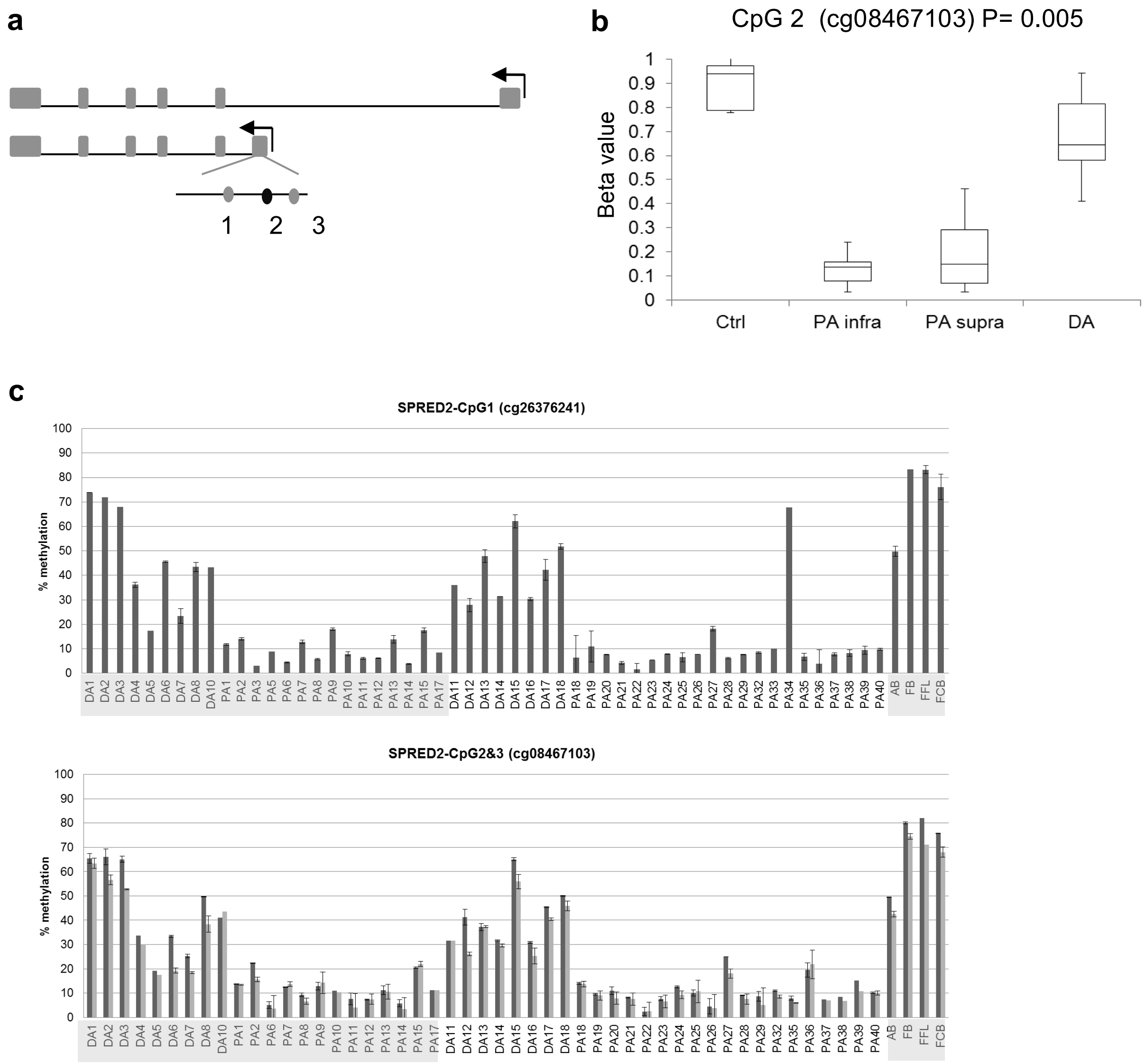

Supplementary Figure 3. Differential methylation of CpG sites within miR-21 in pilocytic and diffuse astrocytomas

(**a**) Diagram showing CpG sites located upstream of the miR-21 gene. (**b**) Probe cg15759721, is significantly hypomethylated in pilocytic astrocytomas compared to diffuse astrocytomas and normal brain. This occurs predominately in infratentorial pilocytic tumours. (**c**) Up-regulation of the miR-21 transcript in pilocytic and some diffuse astrocytomas correlates with expression of *TMEM49* gene. (**d**) Pyrosequencing validation for CpG sites detected by probes cg15759721 and cg02515217 (bisulphite conversion, n=1). DA, diffuse astrocytomas; PA infra, infratentorial pilocytic astrocytomas; PA supra, supratentorial pilocytic astrocytomas; FCB, foetal cerebellum; FD, foetal diencephalon; FCC, foetal cerebral cortex; ACB, adult cerebellum; ACC, adult cerebral cortex; AD, adult diencephalon; AT, adult thalamus; AM, adult medulla; UTR, untranslated region.

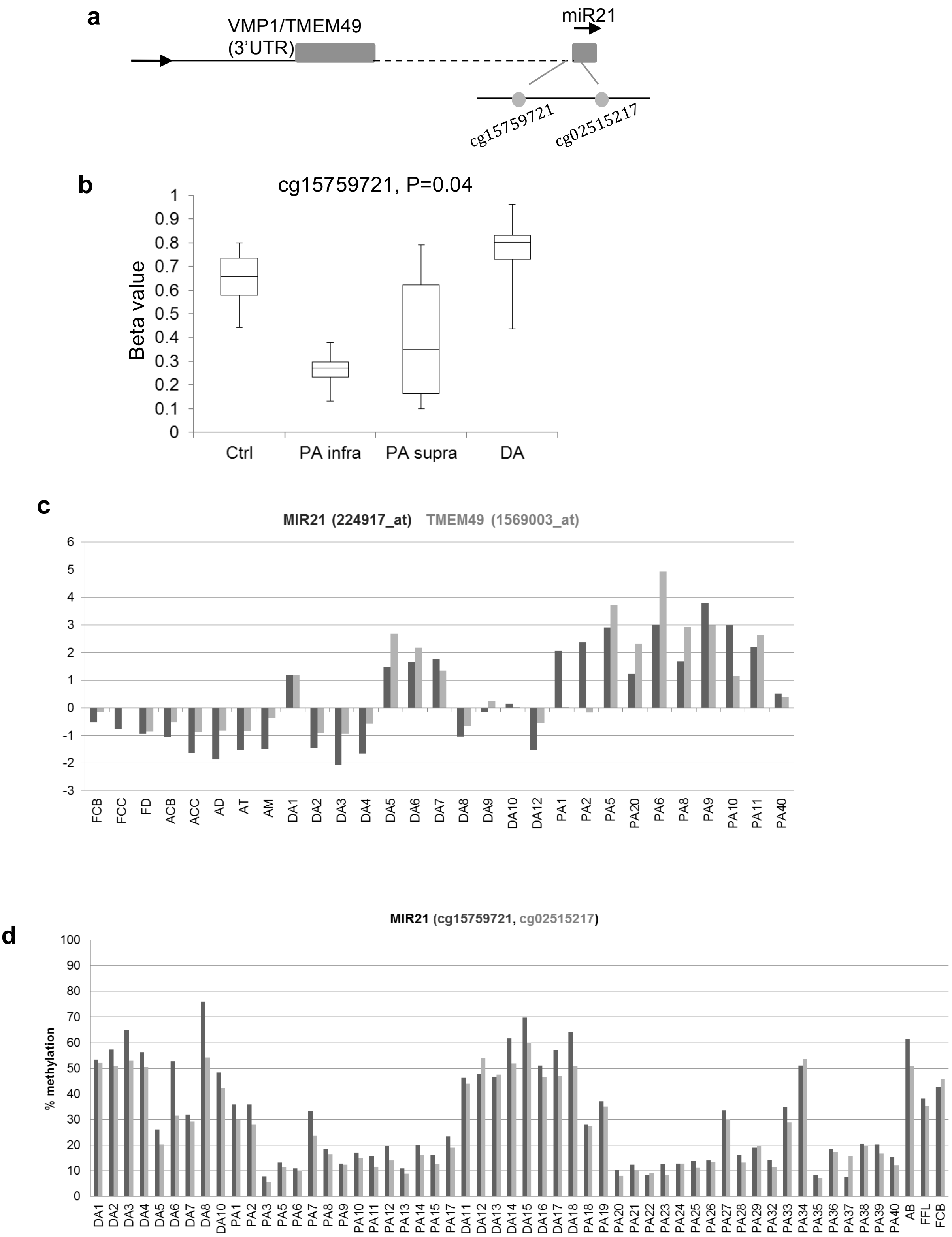
a
VMP1/TMEM49
(3'UTR)
miR21
cg15759721
cg02515217
b
cg15759721, P=0.04
Beta value
1
0.9
0.8
0.7
0.6
0.5
0.4
0.3
0.2
0.1
0
Ctrl
PA infra
PA supra
DA
c
MIR21 (224917_at) TMEM49 (1569003_at)
6
5
4
3
2
1
0
-1
-2
-3
FCB FCC FD ACB ACC AD AT AM DA1 DA2 DA3 DA4 DA5 DA6 DA7 DA8 DA9 DA10 DA12 PA1 PA2 PA5 PA20 PA6 PA8 PA9 PA10 PA11 PA40
d
MIR21 (cg15759721, cg02515217)
% methylation
100
90
80
70
60
50
40
30
20
10
0
DA1 DA2 DA3 DA4 DA5 DA6 DA7 DA8 DA10 PA1 PA2 PA3 PA5 PA6 PA7 PA8 PA9 PA10 PA11 PA12 PA13 PA14 PA15 PA17 DA11 DA12 DA13 DA14 DA15 DA16 DA17 DA18 PA18 PA19 PA20 PA21 PA22 PA23 PA24 PA25 PA26 PA27 PA28 PA29 PA32 PA33 PA34 PA35 PA36 PA37 PA38 PA39 PA40 AB FFL FCB

Supplementary Figure 4. Differentially methylated CpG site within MIR155HG in pilocytic and diffuse astrocytomas

(a) Diagram showing the differentially methylated probe located within the miR-155 gene, which is embedded in the third exon of the MIR155HG host gene [2]. (b) miR-155 probe cg13309012 is significantly hypomethylated in pilocytic astrocytomas compared to diffuse astrocytomas and normal brain. The outliers in the controls are neural stem cells, pilocytic tumours PA6, and diffuse astrocytomas DA5 and DA7 (BRAFv600e). (c) Up regulation of the miR155HG gene in pilocytic and some diffuse astrocytomas. Levels of Affymetrix U133plus2 array probe 229437 are shown in the bar chart. (d) Pyrosequencing validation of the CpG sites detected by probe cg13309012 (bisulphite conversion, n=1). DA, diffuse astrocytomas; PA infra, infratentorial pilocytic astrocytomas; PA supra, supratentorial pilocytic astrocytomas; FCB, foetal cerebellum; FD, foetal diencephalon; FCC, foetal cerebral cortex; ACB, adult cerebellum; ACC, adult cerebral cortex; AD, adult diencephalon; AT, adult thalamus; AM, adult medulla.

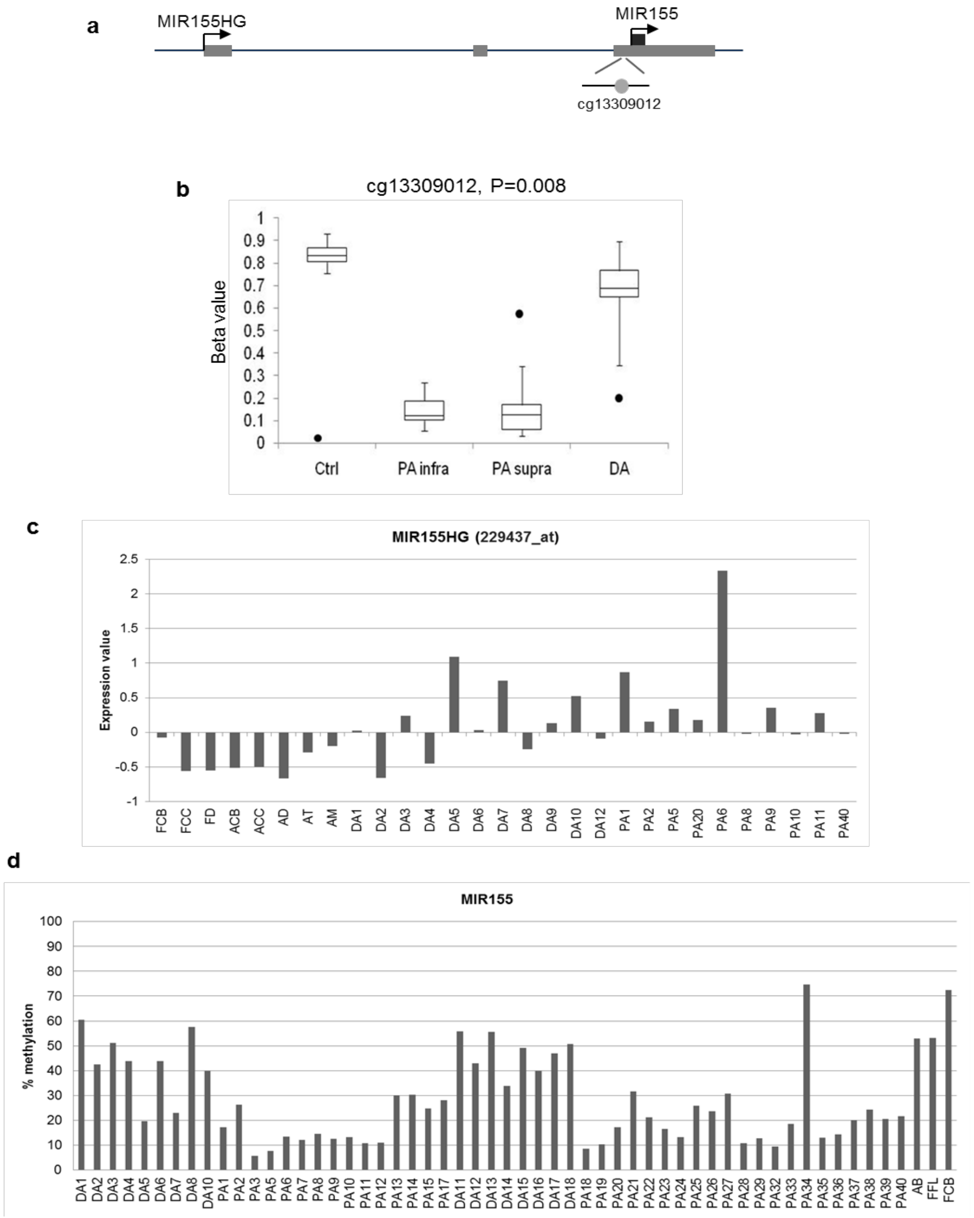

Supplementary Figure 5. Upregulation of miR-21 but not miR-155 in low-grade astrocytomas compared to controls

(a) Expression levels are represented as fold changes from real-time PCR analysis of miR-21 and miR-155. (b) Grouped expression summary shows significant (* P<0.05) up-regulation of miR-21 but not miR-155 in pilocytic and diffuse astrocytomas. DA, diffuse astrocytomas; PA, pilocytic astrocytomas; AB, adult brain; FB, foetal brain; FFL, foetal frontal lobe; FCB, foetal cerebellum.

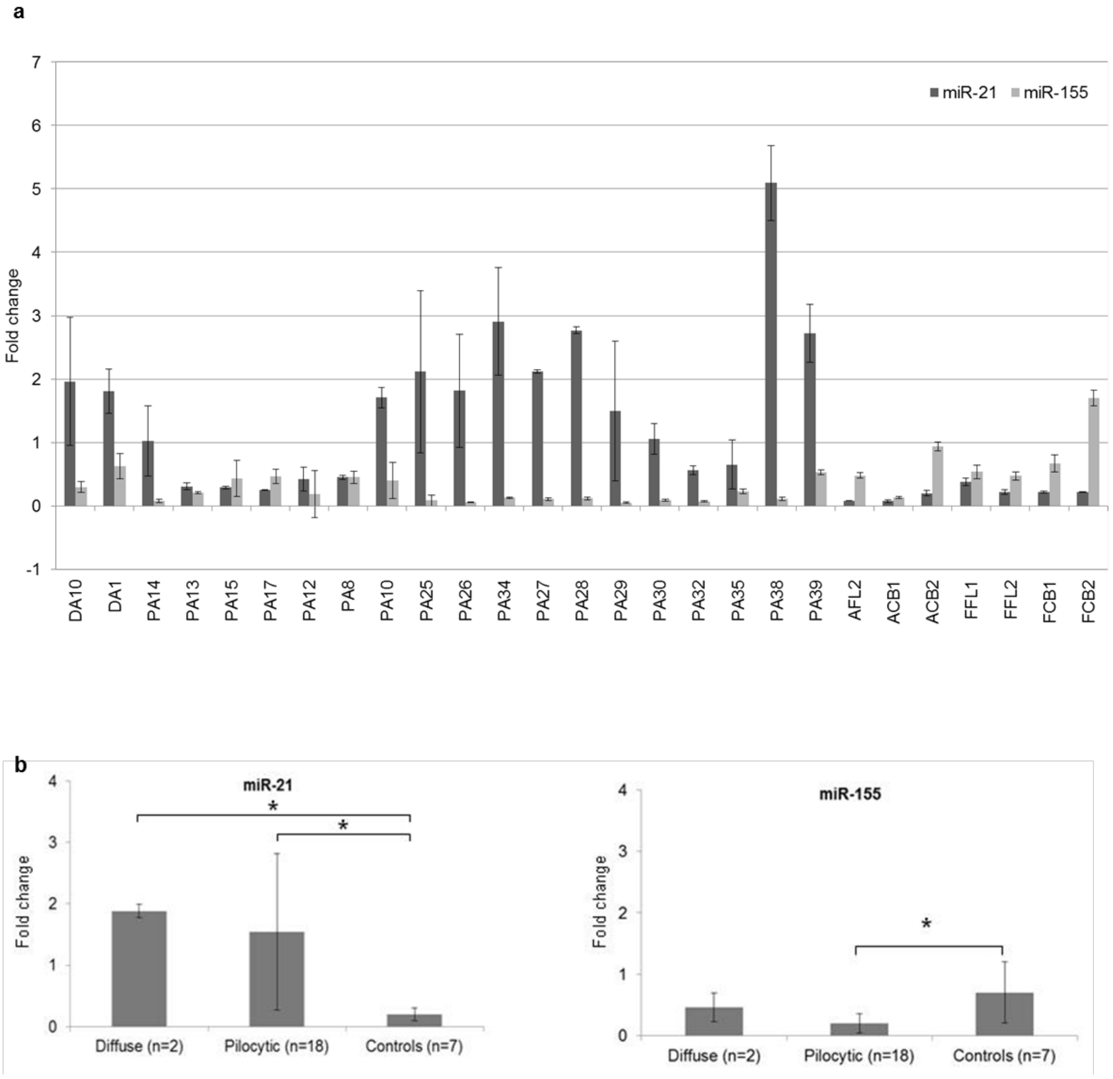

Supplementary Figure 6. Differential methylation in *ABCC3* is linked to gene expression

(a) Diagram showing the CpG probe cg25928474 within the *ABCC3* gene, for the long and shorter transcripts. The probe is located within intron1 of *ABCC3*. (b) Probe cg25928474 is significantly hypomethylated in pilocytic astrocytomas compared to normal brain. (c) *ABCC3* is upregulated in pilocytic and some diffuse astrocytomas. Levels of gene expression for probes 208161_s_at (full transcript without 3'UTR) and 209641 (with 3'UTR) are shown. (d) Validation of cg25928474 for the study and validation tumour sets using pyrosequencing. Error bars shown for technical replicas (two bisulphite conversions, n=2). Correlation analysis is shown in **Supplementary Table 15**. DA, diffuse astrocytomas; PA, pilocytic astrocytomas; FCB, foetal cerebellum; FD, foetal diencephalon; FCC, foetal cerebral cortex; ACB, adult cerebellum; ACC, adult cerebral cortex; AD, adult diencephalon; AT, adult thalamus; AM, adult medulla.

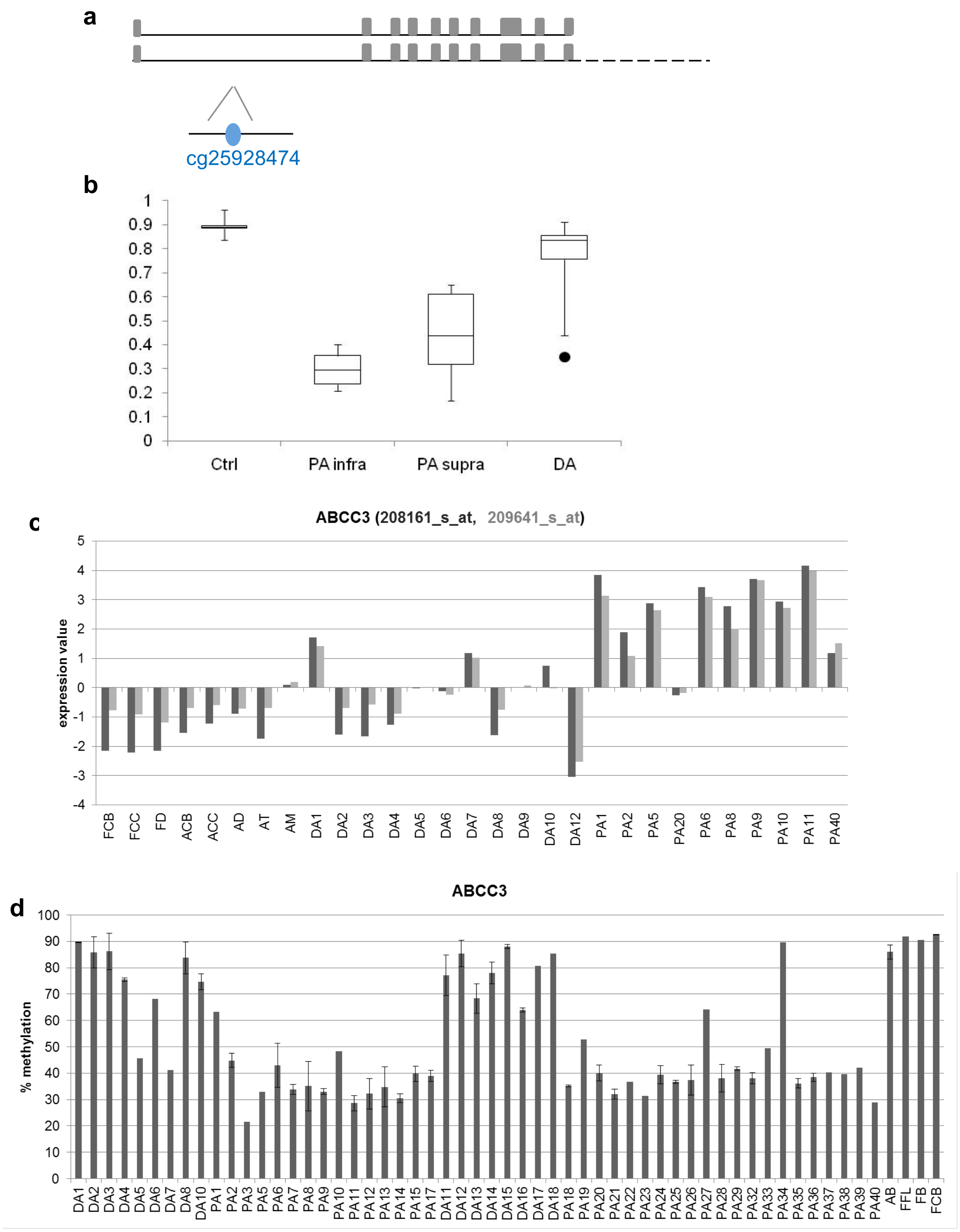
a
cg25928474
b
Ctrl
PA infra
PA supra
DA
c
ABCC3 (208161_s_at, 209641_s_at)
expression value
FCB FCC FD ACB ACC AD AT AM DA1 DA2 DA3 DA4 DA5 DA6 DA7 DA8 DA9 DA10 DA12 PA1 PA2 PA5 PA20 PA6 PA8 PA9 PA10 PA11 PA40
d
ABCC3
% methylation
DA1 DA2 DA3 DA4 DA5 DA6 DA7 DA8 DA10 PA1 PA2 PA3 PA5 PA6 PA7 PA8 PA9 PA10 PA11 PA12 PA13 PA14 PA15 PA17 DA11 DA12 DA13 DA14 DA15 DA16 DA17 DA18 PA18 PA19 PA20 PA21 PA22 PA23 PA24 PA25 PA26 PA27 PA28 PA29 PA32 PA33 PA34 PA35 PA36 PA37 PA38 PA39 PA40 AB FFL FB FCB

Supplementary Figure 7. Methylation of the *SELENBP1* promoter region in low-grade astrocytomas

(a) Diagram showing the differentially methylated region within the *SELENBP1* gene. Probes are located within a CpG island in the promoter region, with all 3 probes showing significant differential methylation (p-value <0.05). (b) *SELENBP1* probe cg24480379 is significantly methylated in pilocytic astrocytomas and diffuse astrocytomas compared to normal brain. (c) *SELENBP1* is down regulated in pilocytic and some diffuse astrocytomas compared to adult brain regions. Levels of gene expression (probe 214433_s_at) are shown. (d) Pyrosequencing validation of CpG probe cg24480379 and surrounding 2 CpG sites, for the study and validation tumour sets. DA, diffuse astrocytomas; PA, pilocytic astrocytomas; FCB, foetal cerebellum; FD, foetal diencephalon; FCC, foetal cerebral cortex; ACB, adult cerebellum; ACC, adult cerebral cortex; AD, adult diencephalon; AT, adult thalamus; AM, adult medulla.

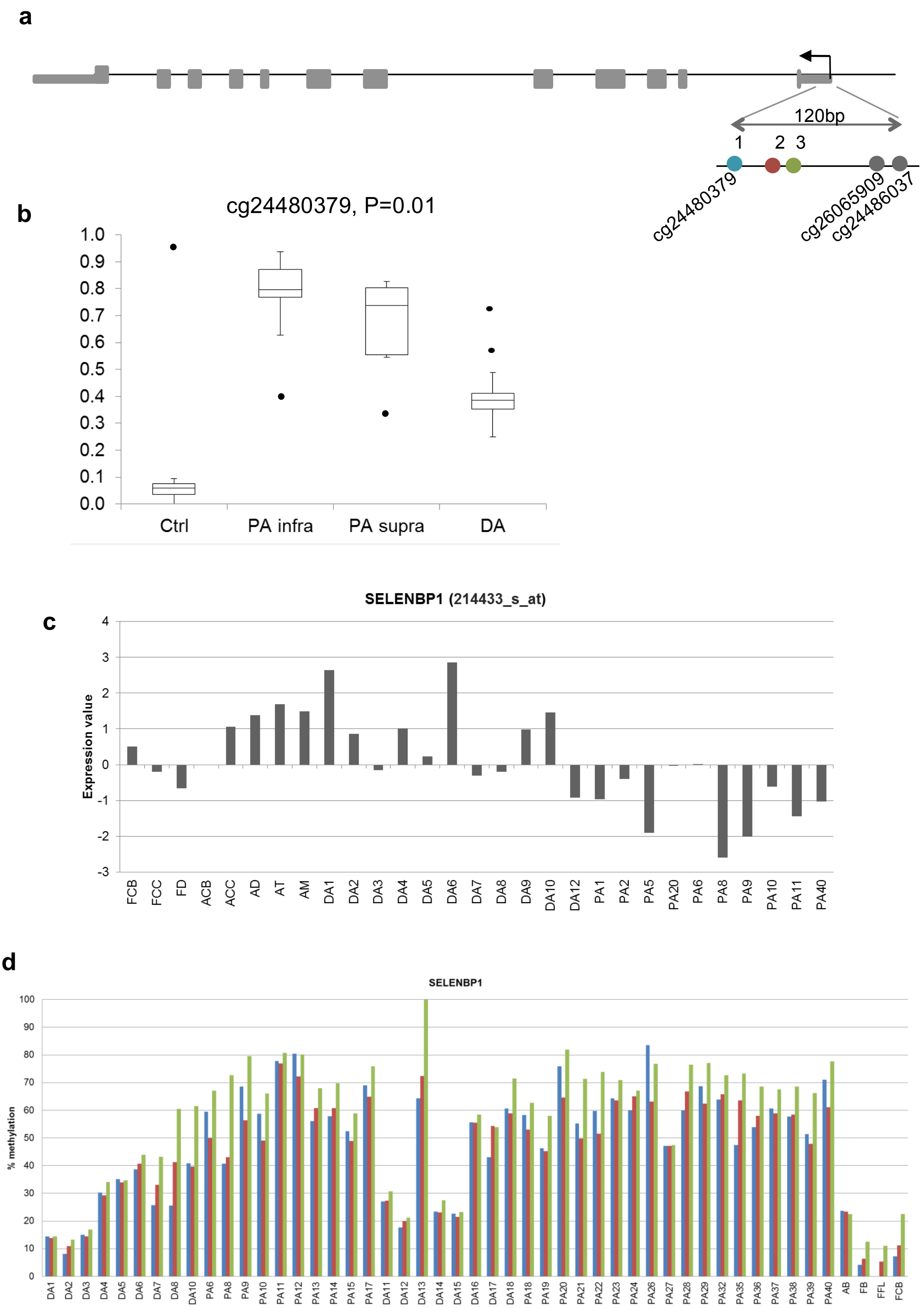

a
120bp
1
2
3
cg24480379
cg26065909
cg24486037
b
cg24480379, P=0.01
1.0
0.9
0.8
0.7
0.6
0.5
0.4
0.3
0.2
0.1
0.0
Ctrl
PA infra
PA supra
DA
c
SELENBP1 (214433_s_at)
Expression value
4
3
2
1
0
-1
-2
-3
FCB FCC FD ACB ACC AD AT AM DA1 DA2 DA3 DA4 DA5 DA6 DA7 DA8 DA9 DA10 DA12 PA1 PA2 PA5 PA20 PA6 PA8 PA9 PA10 PA11 PA40
d
SELENBP1
% methylation
100
90
80
70
60
50
40
30
20
10
0
DA1 DA2 DA3 DA4 DA5 DA6 DA7 DA8 DA10 PA6 PA8 PA9 PA10 PA11 PA12 PA13 PA14 PA15 PA17 DA11 DA12 DA13 DA14 DA15 DA16 DA17 DA18 PA18 PA19 PA20 PA21 PA22 PA23 PA24 PA26 PA27 PA28 PA29 PA32 PA35 PA36 PA37 PA38 PA39 PA40 AB FB FFL FCB

Supplementary Figure 8. Comparison of the hypomethylation signature identified in pilocytic astrocytomas with methylation in a validation tumour set

Heatmap showing the hypomethylation signature in the original test tumour set and in validation tumour set 1, which consisted of 59 low-grade astrocytomas analysed independently using the Illumina Infinium HumanMethylation450 BeadChip system. As 11 values were missing from the validation set 1 dataset, only 304/315 sites are shown. CpG sites within genes of interest are shown (genes shown in red – inflammation, brown – astrocytic and oligodendrocyte markers, blue – tumour suppressors, purple – downstream targets of MAPK pathway, green – receptors, black – others). Tumour samples left of the red line are the original test tumour set, samples right of the line are the validation set. Columns represent individual tumour samples; rows represent 450K CpG probes. Methylation beta values are represented in shades of yellow = 0 (unmethylated) to blue = 1 (methylated).

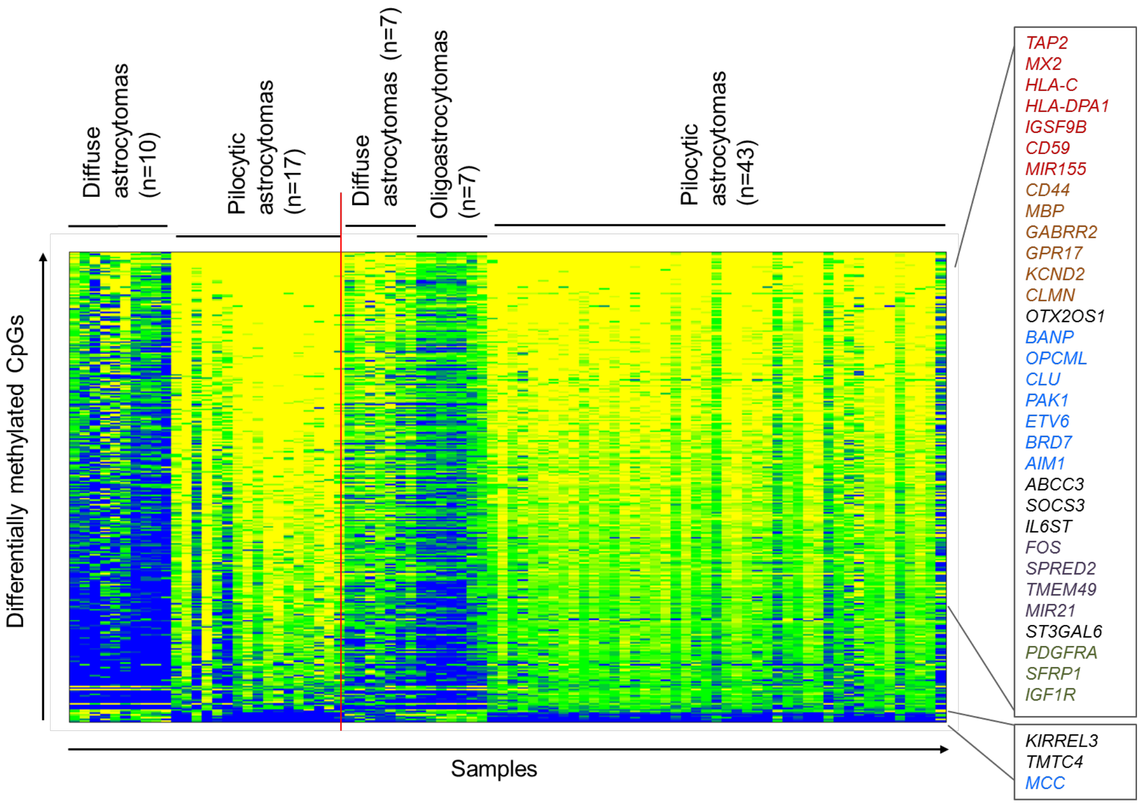

Supplementary Figure 9. Comparison of the hypomethylation signature identified in pilocytic astrocytomas with methylation in an adult low-grade astrocytoma cohort and a high-grade glioma cohort

Heatmaps showing the hypomethylation signature in **(a)** Adult low-grade astrocytoma cohort (TCGA) and **(b)** Adult and paediatric glioblastoma cohort (GSE36278, [10]). Columns represent individual samples; rows 311 CpG probes (Supplementary Table 18). Methylation beta values are represented in shades of yellow = 0 (unmethylated) to blue = 1 (methylated). Study test tumours are shown, pilocytic (n=17) and diffuse (n=10) astrocytomas left of the red line. (Supplementary Table 8).

a

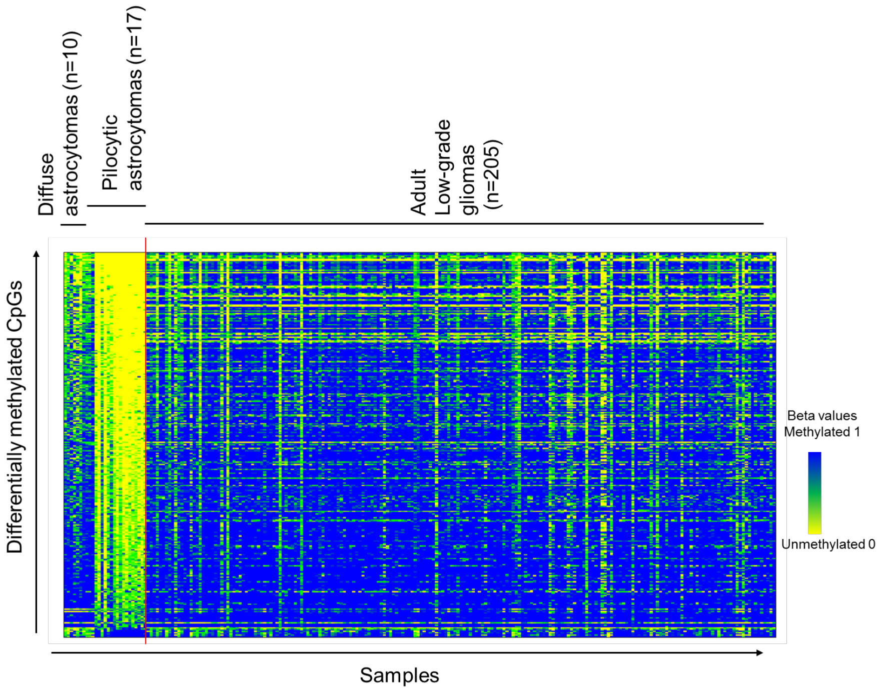

b

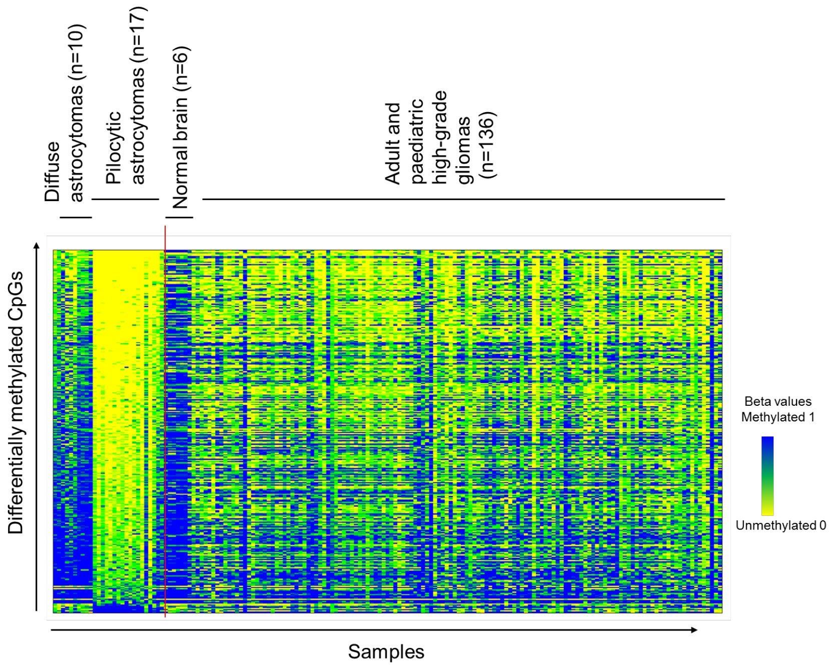

Supplementary Figure 10. Comparison of the hypomethylation signature identified in pilocytic astrocytomas with methylation in a medulloblastoma and an ependymoma cohort

Heatmap showing the hypomethylation signature in **(a)** Medulloblastoma cohort (n=60, grouped according to their molecular status; [8]) and **(b)** Ependymoma cohort (n=36); [6]. Columns represent individual samples; rows represent 450K CpG probes. Methylation beta values are represented in shades of yellow = 0 (unmethylated) to blue =1 (methylated). Study test tumours are shown, pilocytic (n=17) and diffuse (n=10) astrocytomas. As values were missing from the cohorts, 291/315 CpG sites for medulloblastoma and 295/315 CpG sites for ependymomas are shown (Supplementary Table 8).

a

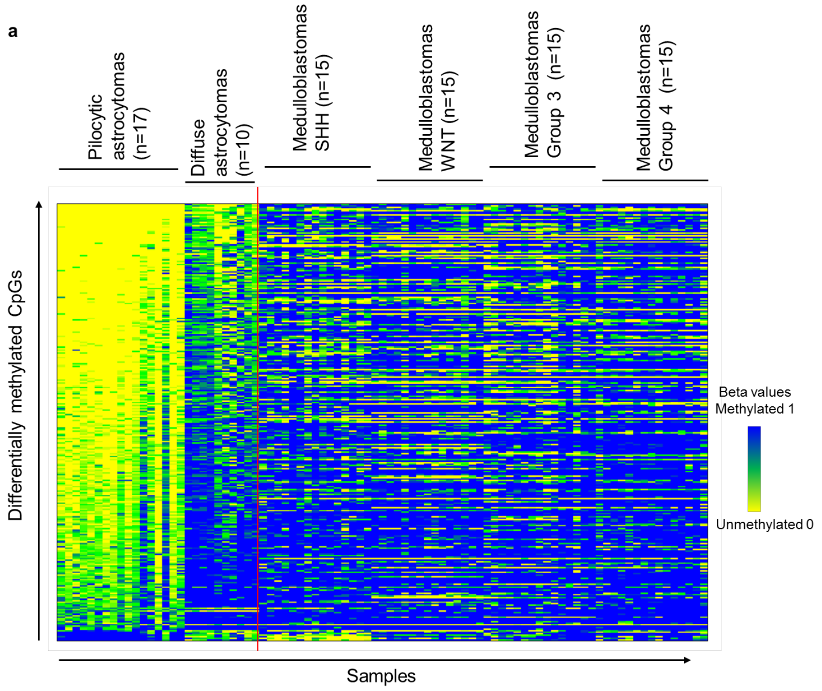

b

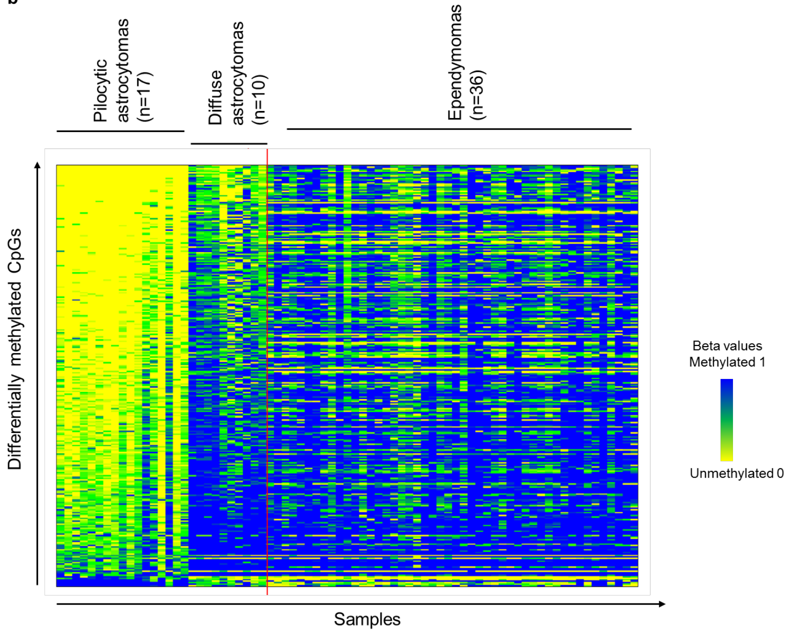

Supplementary Figure 11. Comparison of the hypomethylation signature identified in pilocytic astrocytomas with a foetal brain cohort

Heatmap showing the hypomethylation signature in foetal brain cohort (n=179) representing different stages of brain development from 23dpc-184dpc (GSE58885 [9]. Columns represent individual samples; rows represent 450K CpG probes. Methylation beta values are represented in shades of yellow = 0 (unmethylated) to blue =1 (methylated). Study test tumours are shown, pilocytic (n=17) and diffuse (n=10) astrocytomas left of red line. As values were missing from the cohort, 287/315 CpG sites are shown (Supplementary Table 8).

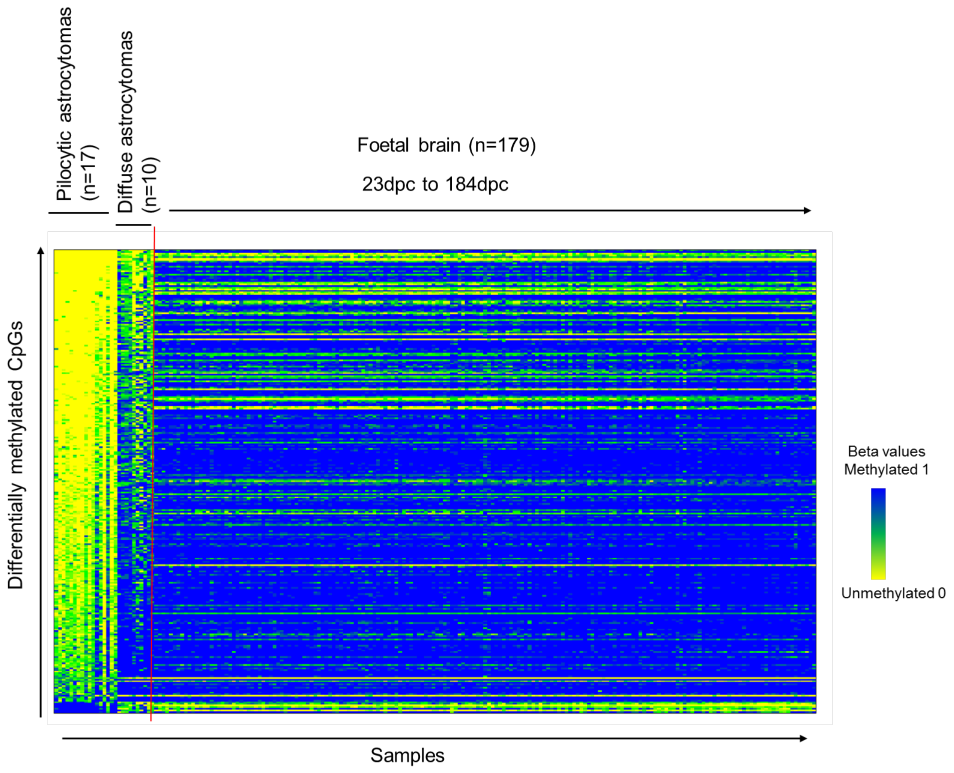

Supplementary Figure 12. Expression of the AP-1 factors of the FOS and JUN families.

Expression levels of AP-1 factors in normal brain controls (Foetal controls – diencephalon, cerebellum, cerebral cortex; Adult controls – diencephalon, medulla, thalamus, cerebellum, cerebral cortex), diffuse astrocytomas and pilocytic astrocytomas. Affymetrix probe identifiers are shown (** p-value <0.01, * p-value <0.05, Mann- Whitney U-test).

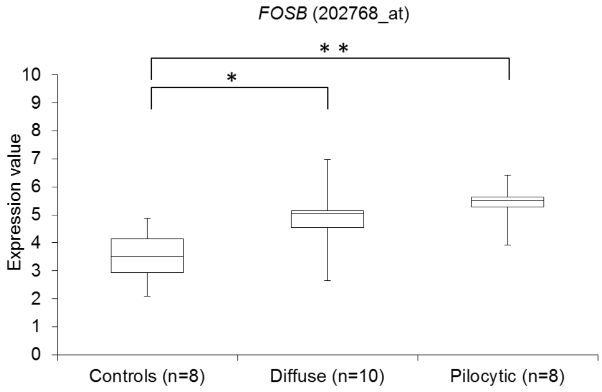

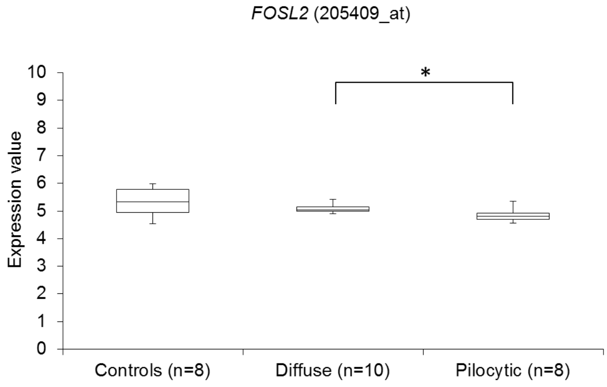

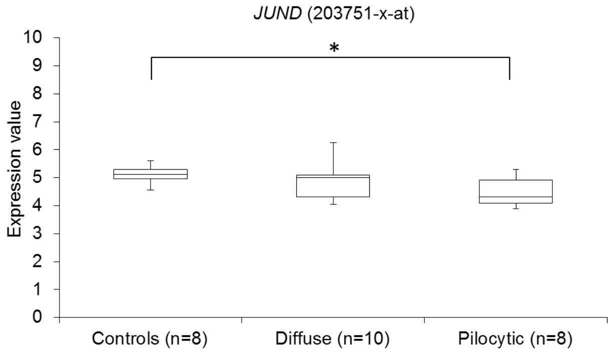

Supplementary Figure 13. Differential methylation within the *CCND1* gene and comparison with SNP status and levels of gene expression.

(a) A 372bp region covering all of exon 4 and 224bp of intron 4 was Sanger-sequenced to identify the methylation status of the 11 CpG sites located within this region (**a.i**). Qualitative methylation status was assigned to each CpG site for 4 normal brain controls, 9 diffuse astrocytomas and 23 pilocytic astrocytomas (**a.ii**). SNP G870A is located at CpG1. Probe cg27021553 detects the 1st CpG site within exon 4. The CpG location of probe cg07295918 is also shown. **(b)** Expression levels of *CCND1a* and *CCND1b* measured by real-time PCR**.** *CCND1a* and *CCND1b* expression in tumours was compared to foetal cerebellum, which had the highest levels of *CCND1b* expression. Normalisation was performed using housekeeping genes *TBP* (**b-i**) and *GAPDH* (**b-ii**). Error bars show standard deviations between cDNA conversions (n=2 for *TBP*; n=1 for *GAPDH*). **(c)** Pyrosequencing analysis of methylation at the G870A SNP site (CpG1) and intronic CpG site (CpG2) for individual tumours and controls, and comparison with SNP status. Error bars are the standard deviation between the averaged values for the bisulphite conversions (n=2). Methylation levels did not correlate with SNP status (CpG1, Pearson correlation -0.33, p-value = 0.07; CpG2, Pearson correlation -0.02, p-value = 0.93). No bias in allele status was seen in the low-grade astrocytomas when compared to dbSNP population allele frequencies, A-allele 41.35% and G-allele 58.65% (Chi square p-values >0.1 for both alleles). **(d)** Summary of *CCND1a* and *CCND1b* expression, methylation and SNP status for each tumour. Significant positive correlation was identified between expression of the two variants (Pearson coefficient 0.51, p-value = 0.013). No significant correlation was identified between *CCND1* expression and methylation status at CpG2 (*CCND1a*, Pearson coefficient -0.09, p-value = 0.69; *CCND1b*, Pearson coefficient 0.38, p-value = 0.08). No correlation was also shown between *CCND1b* expression and SNP status (Pearson coefficient -0.26, p=0.23) or SNP status and methylation status at CpG2 (CpG2, Pearson coefficient -0.33, p-value = 0.069).

a

i

ii

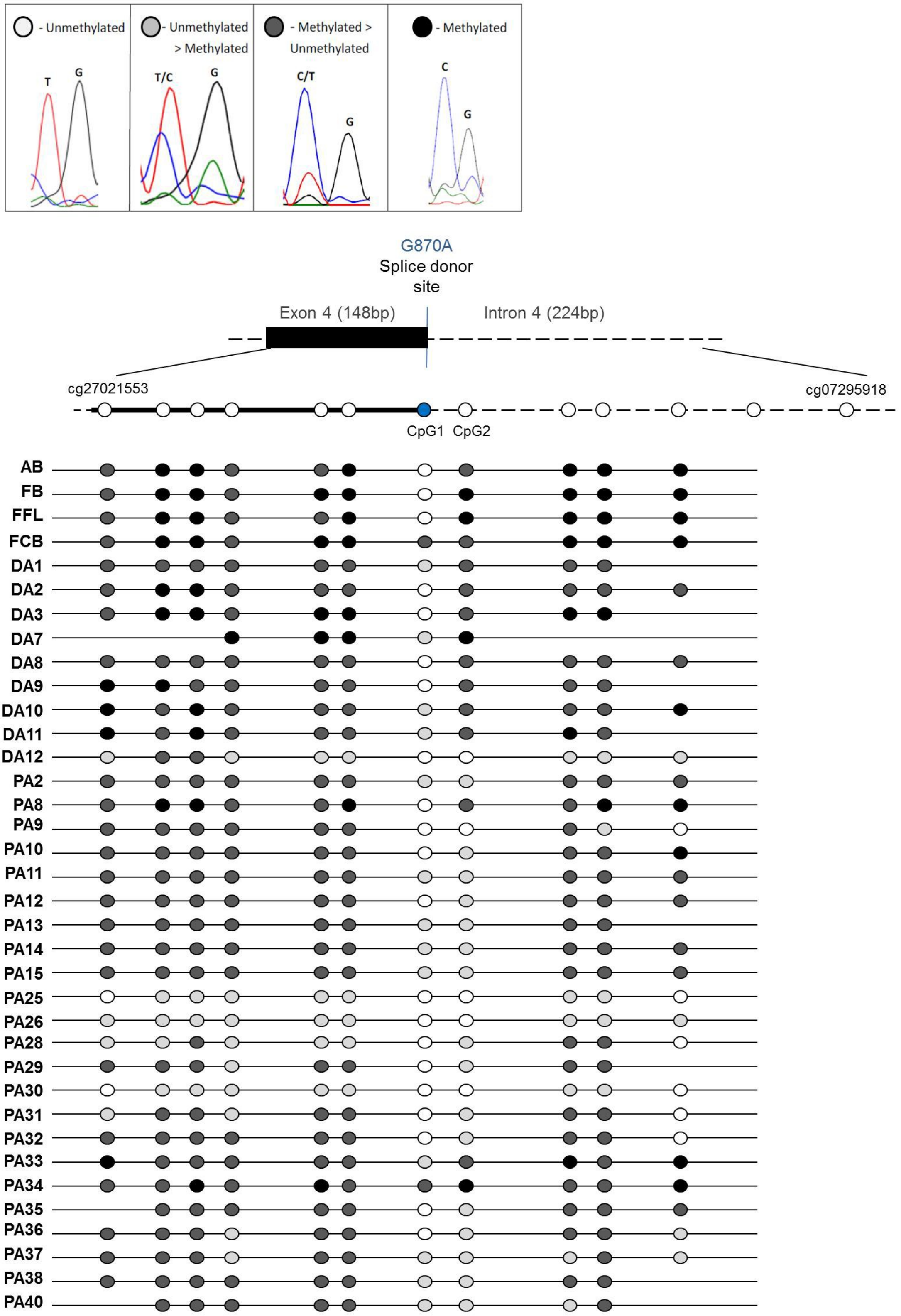
- Unmethylated
- Unmethylated > Methylated
- Methylated > Unmethylated
- Methylated
T
G
T/C
G
C/T
G
C
G
G870A
Splice donor site
Exon 4 (148bp)
Intron 4 (224bp)
cg27021553
cg07295918
CpG1
CpG2
AB
FB
FFL
FCB
DA1
DA2
DA3
DA7
DA8
DA9
DA10
DA11
DA12
PA2
PA8
PA9
PA10
PA11
PA12
PA13
PA14
PA15
PA25
PA26
PA28
PA29
PA30
PA31
PA32
PA33
PA34
PA35
PA36
PA37
PA38
PA40

b

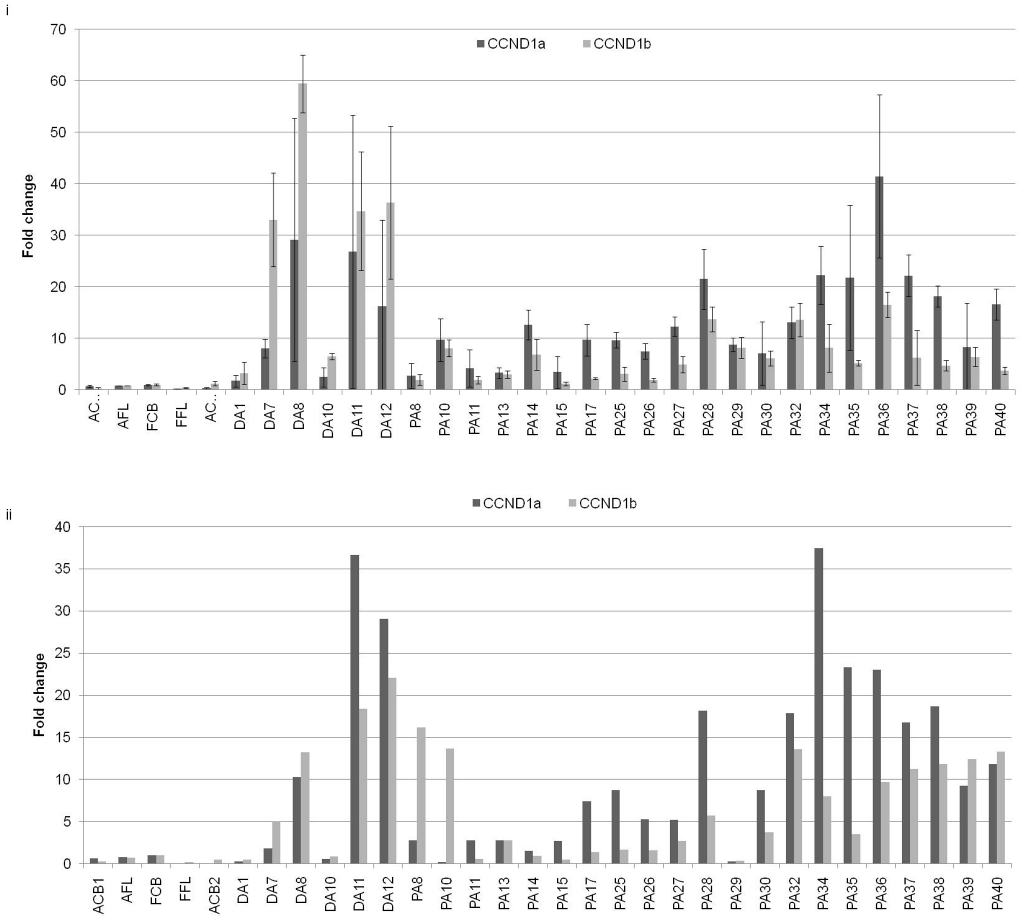
i
CCND1a
CCND1b
Fold change
70
60
50
40
30
20
10
0
AC..
AFL
FCB
FFL
AC..
DA1
DA7
DA8
DA10
DA11
DA12
PA8
PA10
PA11
PA13
PA14
PA15
PA17
PA25
PA26
PA27
PA28
PA29
PA30
PA32
PA34
PA35
PA36
PA37
PA38
PA39
PA40
ii
CCND1a
CCND1b
Fold change
40
35
30
25
20
15
10
5
0
ACB1
AFL
FCB
FFL
ACB2
DA1
DA7
DA8
DA10
DA11
DA12
PA8
PA10
PA11
PA13
PA14
PA15
PA17
PA25
PA26
PA27
PA28
PA29
PA30
PA32
PA34
PA35
PA36
PA37
PA38
PA39
PA40

c

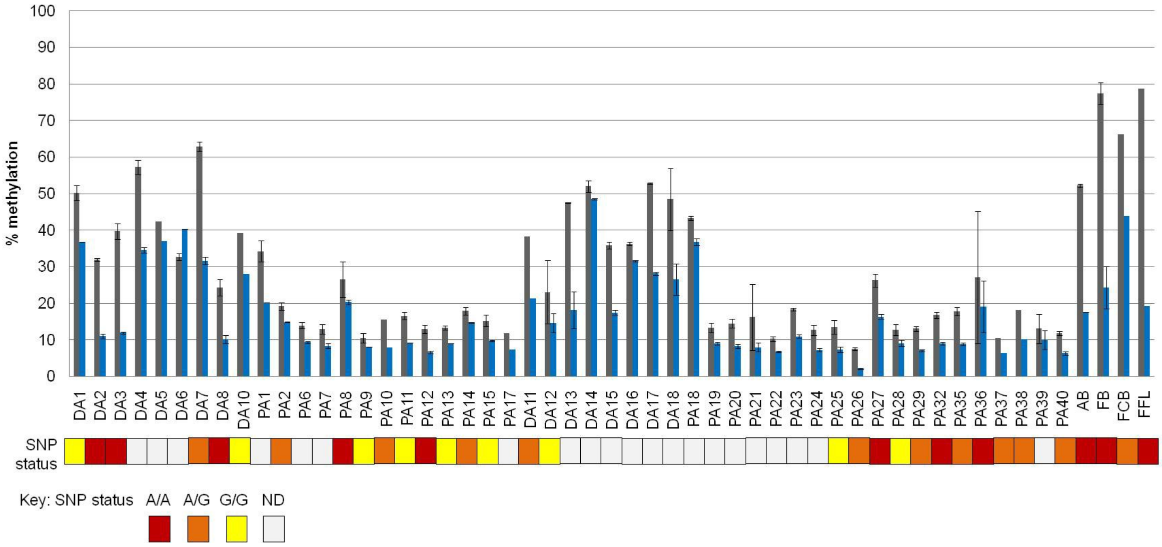
% methylation
100
90
80
70
60
50
40
30
20
10
0
DA1
DA2
DA3
DA4
DA5
DA6
DA7
DA8
DA10
PA1
PA2
PA6
PA7
PA8
PA9
PA10
PA11
PA12
PA13
PA14
PA15
PA17
DA11
DA12
DA13
DA14
DA15
DA16
DA17
DA18
PA18
PA19
PA20
PA21
PA22
PA23
PA24
PA25
PA26
PA27
PA28
PA29
PA32
PA35
PA36
PA37
PA38
PA39
PA40
AB
FB
FCB
FFL
SNP status
Key: SNP status A/A A/G G/G ND

d

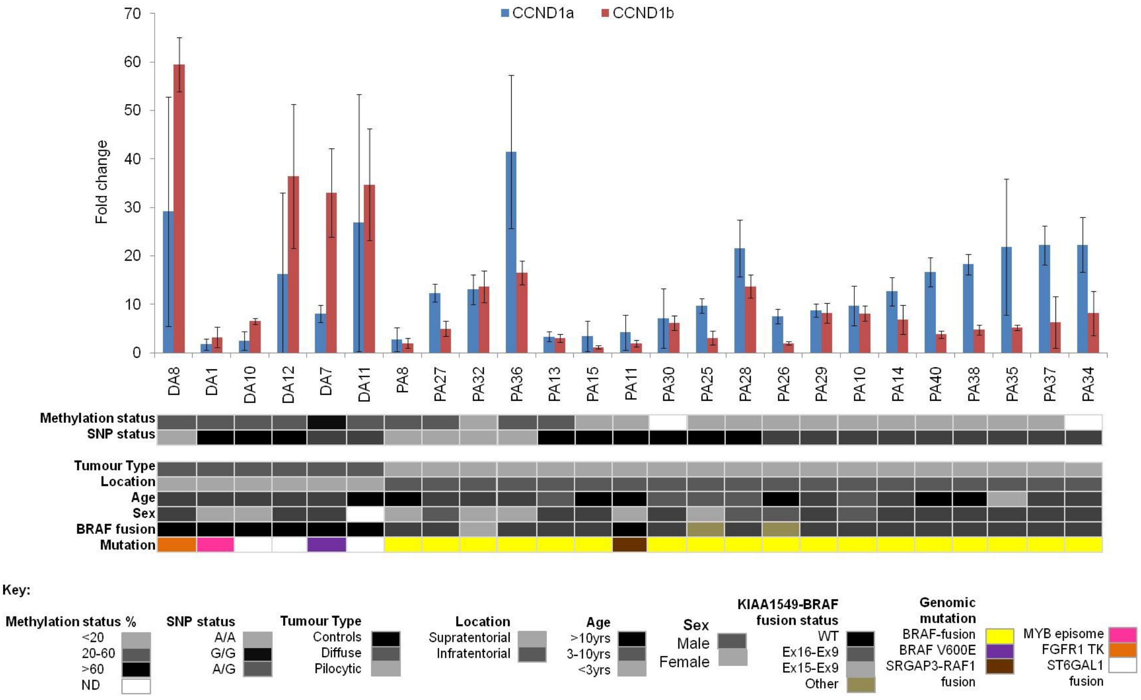
CCND1a
CCND1b
Fold change
70
60
50
40
30
20
10
0
DA8
DA1
DA10
DA12
DA7
DA11
PA8
PA27
PA32
PA36
PA13
PA15
PA11
PA30
PA25
PA28
PA26
PA29
PA10
PA14
PA40
PA38
PA35
PA37
PA34
Methylation status
SNP status
Tumour Type
Location
Age
Sex
BRAF fusion
Mutation
Key:
Methylation status %
<20
20-60
>60
ND
SNP status
A/A
G/G
A/G
Tumour Type
Controls
Diffuse
Pilocytic
Location
Supratentorial
Infratentorial
Age
>10yrs
3-10yrs
<3yrs
Sex
Male
Female
KIAA1549-BRAF fusion status
WT
Ex16-Ex9
Ex15-Ex9
Other
Genomic mutation
BRAF-fusion
BRAF V600E
SRGAP3-RAF1 fusion
MYB episome
FGFR1 TK
ST6GAL1 fusion

Supplementary Figure 14. Features of the differentially methylated CpG sites in infratentorial pilocytic astrocytomas and normal cerebellum

(**a**) Distribution of 11,671 differentially methylated CpG sites across annotated gene regions. TSS1500, 201-1500bp upstream of transcription start (TSS); TSS200, up to 200bp upstream of the TSS; UTR, untranslated region. **(b)** Distribution of the differentially methylated CpG sites in relation to CpG density [7]. HC, high CpG density; IC, intermediate CpG density; IC shore, intermediate CpG density shore; LC, low CpG density. PA, pilocytic astrocytomas (n=11); controls, Adult (n=2) and foetal (n=5) cerebellum.

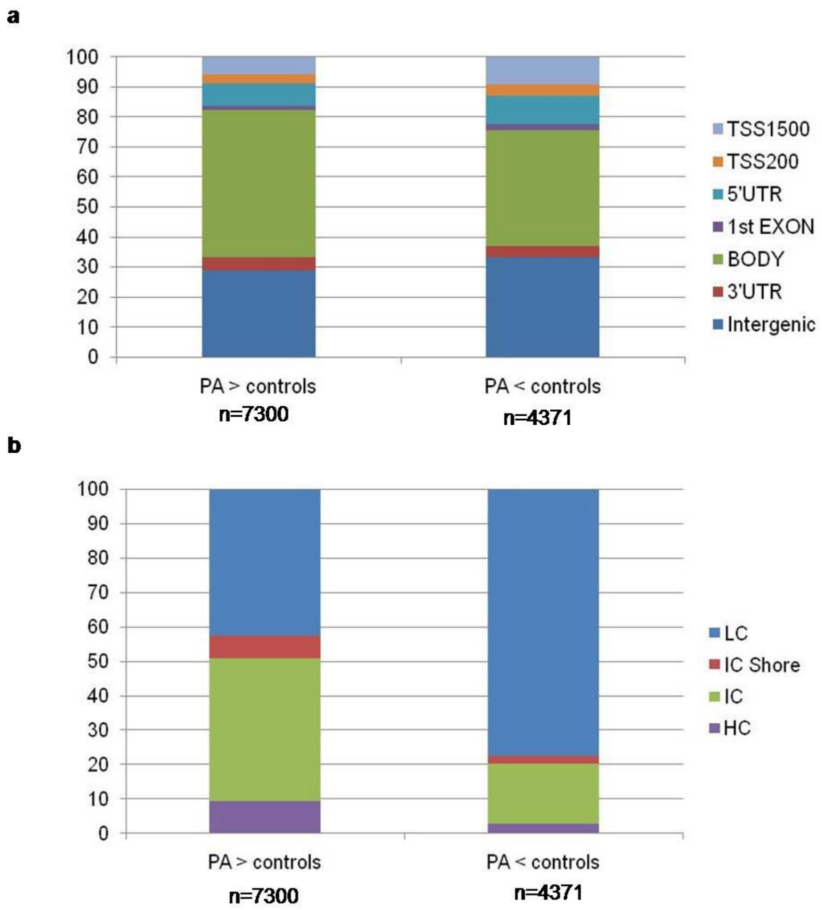

Supplementary Figure 15. Features of the differentially methylated CpG sites in supratentorial astrocytomas and normal cerebral cortex

a) Distribution of differentially methylated CpG sites (382 and 58 sites for pilocytic and diffuse astrocytomas, respectively) across annotated gene regions. **(b)** Distribution of the differentially methylated CpG sites in relation to CpG density [7]. **(c)** Hierarchical clustering of tumours for the 397 CpG sites, 43 of which were common to both tumour types (heatmap). The two major branches separate the normal brain controls from the supratentorial tumours. Pilocytic and diffuse astrocytomas also cluster into separate groups with the exception of PA4. TSS1500, 201-1500bp upstream of transcription start (TSS); TSS200, up to 200bp upstream of the TSS; UTR, untranslated region; HC, high CpG density; IC, intermediate CpG density; IC shore, intermediate CpG density shore; LC, low CpG density. PA, pilocytic astrocytomas (n=6), DA, diffuse astrocytomas (n=10); FFL, Foetal frontal lobe; FB, foetal brain; FCB, foetal cerebellum; AB, adult brain, ACB, adult cerebellum; NSC, neural stem cells; 4782N, 165N, 1230N, 1497N, neuronal component of cerebral cortex from patients aged 18, 15, 16 and 23 years respectively; 4782G, 165G, 1230G, 1497G, glial component of cerebral cortex; MIX1 and MIX7, mix of glial and neuronal components from cerebral cortex of patient aged 50 years, all data from GSE41826 [3].

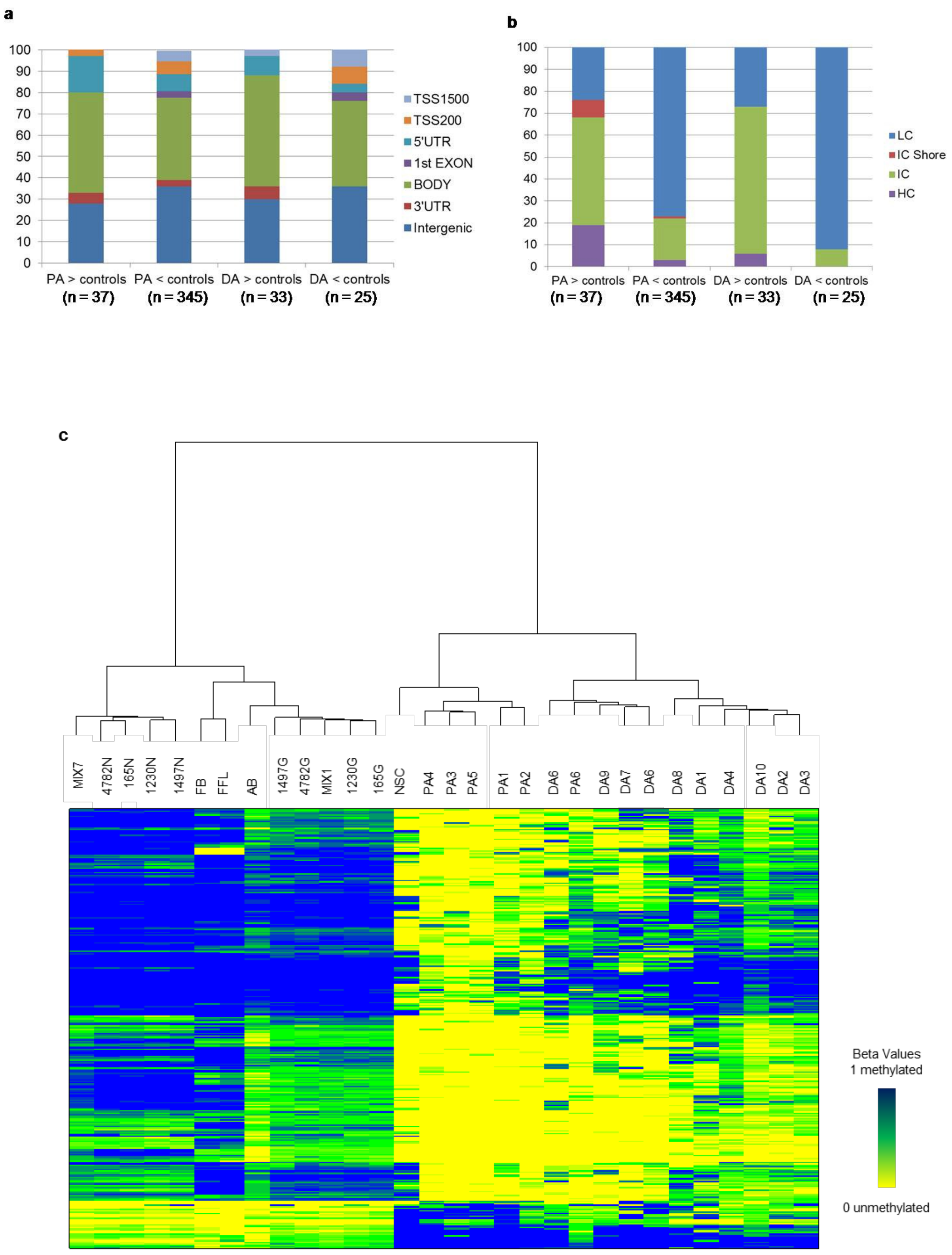
a
100
90
80
70
60
50
40
30
20
10
0
TSS1500
TSS200
5'UTR
1st EXON
BODY
3'UTR
Intergenic
PA > controls (n = 37)
PA < controls (n = 345)
DA > controls (n = 33)
DA < controls (n = 25)
b
LC
IC Shore
IC
HC
PA > controls (n = 37)
PA < controls (n = 345)
DA > controls (n = 33)
DA < controls (n = 25)
c
MIX7
4782N
165N
1230N
1497N
FB
FFL
AB
1497G
4782G
MIX1
1230G
165G
NSC
PA4
PA3
PA5
PA1
PA2
DA6
PA6
DA9
DA7
DA6
DA8
DA1
DA4
DA10
DA2
DA3
Beta Values
1 methylated
0 unmethylated

Supplementary Figure 16. Differentially methylated CpG sites in *NR2E1* distinguish supratentorial and infratentorial tumours

(a) Diagram showing relevant CpG sites within the *NR2E1* gene. The differentially methylated region, which includes probe cg21762523, distinguishes supratentorial and infratentorial normal brain and tumours, unmethylated in infratentorial locations (red box). Probe cg09236658 (blue box) shows a Cancer-associated Differentially Methylated Region (CDMR), which is differentially methylated in some supratentorial pilocytic and diffuse astrocytomas. **(b)** Methylation status for low-grade astrocytomas and normal brain controls. Boxplots represent the degree of methylation (Beta values 1, methylated and 0, unmethylated). Ctrl, normal brain controls (n=5), foetal cerebellum, foetal brain, foetal frontal lobe, adult brain and neural stem cells; PA infra, infratentorial pilocytic astrocytomas (n=11); PA supra, supratentorial pilocytic astrocytomas (n=6); DA, diffuse astrocytomas (n=10). **(c)** Correlation between gene expression and methylation beta values showed stronger positive correlation between gene expression and the CDMR CpG site (r=0.76), than with promoter CpG (r= -0.35). Expression probe 207443_at (U133plus2 Affymetrix array) and methylation status of promoter CpG (TSS200) and body CpG (CDMR) for 16 tumours [infratentorial pilocytic (n=4), supratentorial pilocytic (n=4) and diffuse astrocytomas (n=10)]. **(d)** Pyrosequencing methylation values (%) validating CDMR probe cg09236658. Test tumour set DA1-DA10 and PA1- PA17; the validation tumour set DA11-DA18 and PA18 – PA40. Bar chart represents % methylation for CpG validation of probe cg09236658. Error bars are shown for technical replicas (two bisulphite conversions) for most samples (n=2). DA, diffuse astrocytomas; PA, pilocytic astrocytomas.

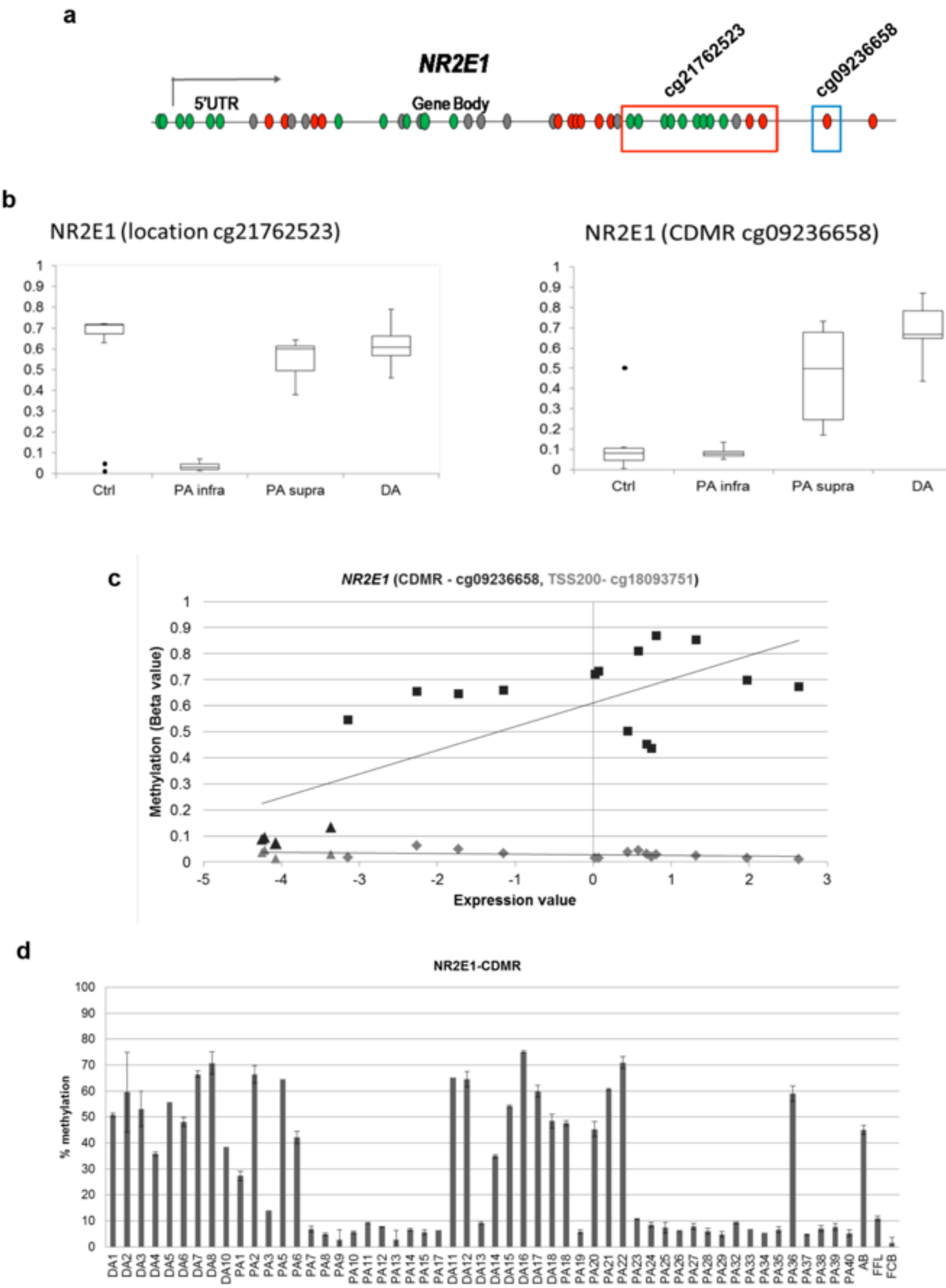
a
NR2E1
5'UTR
Gene Body
cg21762523
cg09236658
b
NR2E1 (location cg21762523)
NR2E1 (CDMR cg09236658)
Ctrl
PA infra
PA supra
DA
c
NR2E1 (CDMR - cg09236658, TSS200- cg18093751)
Methylation (Beta value)
Expression value
d
NR2E1-CDMR
% methylation

Supplementary Figure 17. Differentially methylated CpG sites in *EMX2* and *EMX2OS* distinguish infratentorial and supratentorial tumours

(**a**) Diagram showing the CpG sites within the *EMX2* and *EMX2OS* genes. The gene regions are shown with CpG probes, for the whole genes, represented by numbers. The heatmap show the beta values for the CpG sites for pilocytic and diffuse astrocytomas that have expression data (beta values yellow, unmethylated 0 – blue, methylated 1). The CpG sites are numbered as shown in the gene region. Black asterisk - differentially methylated CpG sites in diffuse astrocytomas only, red asterisk – differentially methylated CpG sites in supratentorial astrocytomas. **(b)** Expression values for the pilocytic astrocytomas, diffuse astrocytomas and normal brain controls. sPA, supratentorial pilocytic astrocytomas (n=4); iPA, infratentorial pilocytic astrocytomas (n=4); DA, diffuse astrocytomas (n=10). Normal controls are not matched samples, expression values are from a single sample. The methylation values are from averaged methylation status – ACC, adult cerebral cortex (n=2); ACB, adult cerebellum (n=2); FCC, foetal cerebral cortex (n=1); foetal cerebellum (n=5).

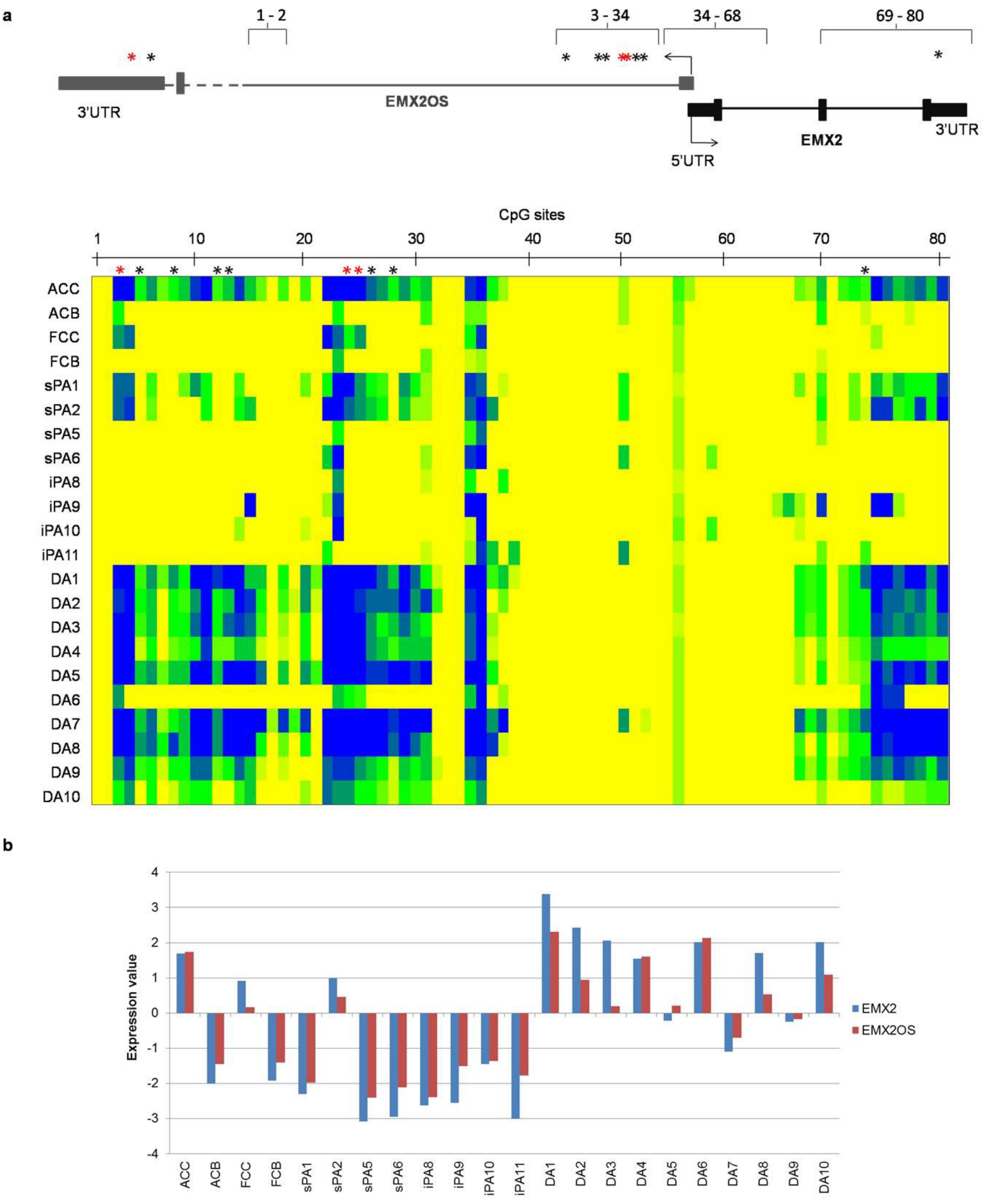
a
1 - 2
3 - 34
34 - 68
69 - 80
3'UTR
EMX2OS
5'UTR
EMX2
3'UTR
CpG sites
ACC
ACB
FCC
FCB
sPA1
sPA2
sPA5
sPA6
iPA8
iPA9
iPA10
iPA11
DA1
DA2
DA3
DA4
DA5
DA6
DA7
DA8
DA9
DA10
b
Expression value
EMX2
EMX2OS

REFERENCES

1 Bibikova M, Barnes B, Tsan C, Ho V, Klotzle B, Le JM, Delano D, Zhang L, Schroth GP, Gunderson KL et al (2011) High density DNA methylation array with single CpG site resolution. Genomics 98: 288-295 Doi 10.1016/j.ygeno.2011.07.007

2 Elton TS, Selemon H, Elton SM, Parinandi NL (2013) Regulation of the MIR155 host gene in physiological and pathological processes. Gene 532: 1-12 Doi 10.1016/j.gene.2012.12.009

3 Guintivano J, Aryee MJ, Kaminsky ZA (2013) A cell epigenotype specific model for the correction of brain cellular heterogeneity bias and its application to age, brain region and major depression. Epigenetics 8: 290-302 Doi 10.4161/epi.23924

4 Kilaru V, Barfield RT, Schroeder JW, Smith AK, Conneely KN (2012) MethLAB: a graphical user interface package for the analysis of array-based DNA methylation data. Epigenetics 7: 225-229 Doi 10.4161/epi.7.3.19284

5 Lambert SR, Witt H, Hovestadt V, Zucknick M, Kool M, Pearson DM, Korshunov A, Ryzhova M, Ichimura K, Jabado N et al (2013) Differential expression and methylation of brain developmental genes define location-specific subsets of pilocytic astrocytoma. Acta Neuropathologica 126: 291-301 Doi 10.1007/s00401-013-1124-7

6 Mack SC, Witt H, Piro RM, Gu L, Zuyderduyn S, Stutz AM, Wang X, Gallo M, Garzia L, Zayne K et al (2014) Epigenomic alterations define lethal CIMP-positive ependymomas of infancy. Nature 506: 445-450 Doi 10.1038/nature13108

7 Price ME, Cotton AM, Lam LL, Farre P, Emberly E, Brown CJ, Robinson WP, Kobor MS (2013) Additional annotation enhances potential for biologically-relevant analysis of the Illumina Infinium HumanMethylation450 BeadChip array. Epigenetics & Chromatin 6: 4 Doi 10.1186/1756-8935-6-4

8 Schwalbe EC, Hayden JT, Rogers HA, Miller S, Lindsey JC, Hill RM, Nicholson SL, Kilday JP, Adamowicz-Brice M, Storer L et al (2013) Histologically defined central nervous system primitive neuro-ectodermal tumours (CNS-PNETs) display heterogeneous DNA methylation profiles and show relationships to other paediatric brain tumour types. Acta Neuropathol 126: 943-946 Doi 10.1007/s00401-013-1206-6

9 Spiers H, Hannon E, Schalkwyk LC, Smith R, Wong CC, O'Donovan MC, Bray NJ, Mill J (2015) Methylomic trajectories across human fetal brain development. Genome Research 25: 338-352 Doi 10.1101/gr.180273.114

10 Sturm D, Witt H, Hovestadt V, Khuong-Quang DA, Jones DT, Konermann C, Pfaff E, Tonjes M, Sill M, Bender S et al (2012) Hotspot mutations in H3F3A and IDH1 define distinct epigenetic and biological subgroups of glioblastoma. Cancer Cell 22: 425-437 Doi 10.1016/j.ccr.2012.08.024
