# Supplementary material for: DNA methylation analysis of paediatric low-grade astrocytomas identifies a tumour-specific hypomethylation signature in pilocytic astrocytomas
Source: Acta Neuropathol Commun. 2016 May 27;4:54. doi: 10.1186/s40478-016-0323-6 (PMC4882864; doi:10.1186/s40478-016-0323-6)
Supplement: Additional file 4: Table S3. — Overview of comparisons performed between pilocytic astrocytomas and diffuse astrocytomas, study controls, and normal brain tissue. Differentially methylated CpG sites (δβ ≥0.3, FDR-corrected p-value <0.05) were identified by MethLAB analysis. (PDF 104 kb) [file 40478_2016_323_MOESM4_ESM.pdf]

Table S3

Overview of comparisons performed between pilocytic astrocytomas and diffuse astrocytomas, study controls, and normal brain tissue

Differentially methylated CpG sites (δβ ≥0.3, FDR-corrected p-value <0.05) were identified by MethLAB analysis.

	Comparison	**Differentially methylated CpG sites***		**Total**
	PA[1] v Ctrls[2]	PA>Ctrls: 375	PA<Ctrls: 583	958
**A**	DA[3] v Ctrls	DA>Ctrls: 252	DA<Ctrls: 100	352
	PA v DA	PA>DA: 11	PA<DA: 304	315
	PAi v CB[4]	PAi>CB: 7,309	PAi<CB: 4,362	11,671
	PAi[5] v FCB[6]	PAi>FCB: 14,488	PAi<FCB: 6,991	21,479
**B**	PAi v ACB[7]	PAi>ACB: 22,187	PAi<ACB: 13,012	35,199
	FCB v ACB	FCB>ACB: 10,281	FCB<ACB: 8,226	18,507
	PAs[8] v CC[9]	PAs>CC: 37	PAs<CC: 345	382
**C**	PAs v FCC[10]	PAs>FCC: 14,156	PAs<FCC: 3,130	17,300
	PAs v ACC[11]	PAs>ACC: 1,164	PAs<ACC: 1,154	2,318
	DA v CC	DA>CC: 33	DA<CC: 25	58
**D**	DA v FCC	DA>FCC: 15,891	DA<FCC: 1,834	17,725
	DA v ACC	DA>ACC: 1,329	DA<ACC: 203	1,532
	PAs v Glial[12]	PAs>Glial: 1,128	PAs<Glial: 1,514	2,642
**E**	PAs v Neuronal[13]	PAs>Neuronal: 9,060	PAs<Neuronal: 13,281	22,341
	DA v Glial	DA>Glial: 1,228	DA<Glial: 270	1,498
	DA v Neuronal	DA>Neuronal: 11,514	DA<Neuronal: 10,772	22,286
	PAi v PAs	PAi>PAs: 7	PAi<PAs: 65	72
**F**	PAi v DA	PAi>DA: 17	PAi<DA: 376	393

[*] For each comparison, the no. of CpG sites which are relatively hypo- or hypermethylated is shown.
[1] PA - Pilocytic astrocytomas, n=17
[2] Ctrls - Study controls, n=5; adult brain; foetal brain, foetal frontal lobe; foetal cerebellum; neural stem cells (REN VM cell-line)
[3] DA - Diffuse astrocytomas, n=10
[4] CB - All cerebellum controls, n=7
[5] PAi – Infratentorial Pilocytic astrocytomas, n=11
[6] FCB - Foetal cerebellum, n=5, study FCB x1; FCB x4 GSE44684 [Lambert et al, 2013, Acta Neuropath 126: 291-301]
[7] ACB - Adult cerebellum, n=2, GSE44684 [Lambert et al, 2013, Acta Neuropath 126: 291-301]
[8] PAs – Supratentorial Pilocytic astrocytomas, n=6
[9] CC – All cerebral cortex controls, n=12
[10] FCC - Foetal cortex controls, n=2, study foetal brain and frontal lobe
[11] ACC - Adult cerebral cortex, n=2, GSE41826 [Guintivano et al, 2013, Epigenetics 8: 290-302]
[12] Glial component of cerebral cortex, n=4, GSE41826 [Guintivano et al, 2013, Epigenetics 8: 290-302]
[13] Neuronal component of cerebral cortex, n=4, GSE41826 [Guintivano et al, 2013, Epigenetics 8: 290-302]
