# Supplementary material for: DNA methylation analysis of paediatric low-grade astrocytomas identifies a tumour-specific hypomethylation signature in pilocytic astrocytomas
Source: Acta Neuropathol Commun. 2016 May 27;4:54. doi: 10.1186/s40478-016-0323-6 (PMC4882864; doi:10.1186/s40478-016-0323-6)
Supplement: Additional file 14: Table S13. — Functions of genes which show a significant correlation between DNA methylation and gene expression. The majority of genes were hypomethylated in pilocytic astrocytomas except the genes highlighted in bold, which were hypermethylated in the pilocytic astrocytomas. (PDF 158 kb) [file 40478_2016_323_MOESM14_ESM.pdf]

Table S13. Functions of genes which show a significant correlation between DNA methylation and gene expression.

The majority of genes were hypomethylated in pilocytic astrocytomas except the genes highlighted in bold, which were hypermethylated in the pilocytic astrocytomas.

Gene Symbol	Gene Name	Gene Function
ABCC3	ATP-Binding Cassette, Sub-Family C (CFTR/MRP), Member 3	ATP-binding cassette (ABC) transporters carry molecules across extra- and intracellular membranes. Involved in multi-drug resistance in cancer.
AIM1	Absent in melanoma 1	Beta/gamma-crystallin domain containing protein. Proposed tumour suppressor role in melanoma. Interacts with the cytoskeleton.
CD59	CD59 complement molecule	Regulates complement-mediated cell lysis. Mutations cause CD59 deficiency, leading to hemolytic anaemia and thrombosis, and in turn to cerebral infarction.
FAM167A	Family with sequence similarity 167, member A	Not known
FOS	FBJ murine osteosarcoma viral oncogene homolog	Downstream target of MAPK pathway. Dimerises with JUN proteins forming transcription factor AP-1 which regulates cell proliferation, differentiation and transformation.
HRH1	Histamine receptor H1	Mediates release of catecholamine from adrenal medulla and neurotransmission. Mediates increase in capillary permeability due to contraction of terminal venules.
IGSF9B	Immunoglobulin superfamily, member 9B	Functions in dendrite outgrowth and synapse maturation.
IL6ST	Interleukin 6 signal transducer (gp130, oncostatin M receptor)	Signal transducer used by many cytokines. Functions as part of cytokine receptor complex.
KCND2	Potassium voltage-gated channel, Shal-related subfamily, member 2	Voltage gated potassium channel, so many functions incl: neutransmitter release, neuronal excitability, smooth muscle contraction and cell volume.
KIRREL3	**Kin of IRRE like 3 (Drosophila)**	**Ensures size- and charge-selective ultrafiltration in brain (foetal and adult) and kidney. Mutation is associated with mental retardation.**
LDLRAD3	Low density lipoprotein receptor class A domain containing 3	Low density lipoprotein receptor. May influence amyloid precursor protein processing.
LEPREL1	Leprecan-like 1	Critical in collagen chain assembly and stability by catalysing hydroxylation of proline residues. Mutations are associated with myopia and cataract and vitreoretinal degeneration. Downregulation may play role in breast cancer.
MIR21	MicroRNA 21	Oncomir that targets several tumour suppressors.
OPCML	Opioid binding protein/cell adhesion molecule-like	Located in the plasma membrane and may be opioid receptor. Highly conserved in mammalian species.
PRSS23	Protease, serine, 23	Serine protease. Highly conserved in vertebrates, may have important ovarian function.
RARRES1	Retinoic acid receptor responder (tazarotene induced) 1	Retinoid acid receptor responsive gene. Upregulated by tazarotene and retinoic receptors. Downregulation in prostate cancer caused by CpG island methylation.
SOCS3	Suppressor of cytokine signaling 3	Negative regulator of cytokine signalling. Induced by cytokines including IL6, IL10 and IFN and can bind to JAK2. Mouse studies suggest roles in negative regulation of foetal liver hematopoieis and placental development.
TM4SF1	Transmembrane 4 L six family member 1	Cell surface antigen. Mediates signal transduction events in developmental regulation, activation, growth and motility. Highly expressed in different carcinomas.
TMEM132C	Transmembrane protein 132C	Transmembrane protein.
TMEM49	VMP1 - vacuole membrane protein	Stress induced protein. When overexpressed, promotes

	1	formation of intracellular vacuoles followed by cell death. Important role in formation of cell junctions, involved in cell-cell adhesion.
TRPM8	Transient receptor potential cation channel, subfamily M, member 8	Receptor activated cation channel associated with sensing coolness (of temperature) or menthol.
WSCD1	WSC domain containing 1	Not known
CDK6	Cyclin-dependent kinase 6	Regulates G1-S phase progression by forming complex with CDK6, phosphorylating RB and allowing E2F to transcribe S phase genes.
CHRM3	Cholinergic receptor, muscarinic 3	Mediates various cellular responses including inhibition of adenylate cyclase, breakdown of phosophoinositides and modulation of potassium channels through action of G proteins.
COTL1	Coactosin-like F-actin binding protein 1	Binds to F actin in a calcium independent manner, interacts with 5-lipoxygenase (first committed enzyme in leukotriene biosynthesis).
DUSP5	Dual specificity phosphatase 5	Phosphatase that negatively regulates ERK1.
FAM89A	Family with sequence similarity 89, member A	Not known
GJA1	Gap junction protein, alpha 1, 43kDa	Component of gap junctions. Major component of gap junctions in heart; important for synchronisng heart contractions and in embryonic development. Mutations related to heart malformations and oculodentodigital dysplasia.
GRIA4	Glutamate receptor, ionotropic, AMPA 4	Glutamate receptor that functions as ligand gated ion channel. Important in synaptic transmission.
IGF1R	Insulin-like growth factor 1 receptor	Receptor that binds insulin-like growth factor with tyrosine kinase activity. Insulin like growth factor plays important role in transformation by indirectly activating RAS-MAPK pathway and PI3K-AKT/PKB pathway.
IRX1	**Iroquois homeobox 1**	**Involved in pattern formation in embryo. Tumour suppressor in gastric and head and neck cancers.**
MCC	**Mutated in colorectal cancers**	**Candidate colorectal tumour suppressor thought to negatively regulate cell cycle progression. Suppresses cell proliferation and WNT/β-Catenin pathway in colorectal cancer.**
MDGA2	MAM domain containing glycosylphosphatidylinositol anchor 2	May be involved in cell-cell interactions.
PRSS23	Protease, serine, 23	Encodes a trypsin. Decrease of mRNA found after induced ovulations in mice. May be an important ovarian protease. Highly conserved in vertebrates.
PTPRE	Protein tyrosine phosphatase, receptor type, E	PTPs are signalling molecules that regulate variety of cellular processes including growth and differentiation, mitotic cycle and oncogenic transformation. Studies of the mouse gene suggest regulator roles in RAS related pathways, cytokine induced SATA signalling and activation of voltage gated K+ channels.
STAMBPL1	STAM binding protein-like 1	Zinc metalloprotease that cleaves lys-63'-linked polyubiquitin chains.
TMEM169	Transmembrane protein 169	Not known
